# Biogenic Silver Nanoparticles Can Control *Toxoplasma gondii* Infection in Both Human Trophoblast Cells and Villous Explants

**DOI:** 10.3389/fmicb.2020.623947

**Published:** 2021-01-21

**Authors:** Idessania Nazareth Costa, Mayara Ribeiro, Priscila Silva Franco, Rafaela José da Silva, Thádia Evelyn de Araújo, Iliana Claudia Balga Milián, Luana Carvalho Luz, Pâmela Mendonça Guirelli, Gerson Nakazato, José Roberto Mineo, Tiago W. P. Mineo, Bellisa Freitas Barbosa, Eloisa Amália Vieira Ferro

**Affiliations:** ^1^Laboratory of Immunoparasitology of Neglected Diseases and Cancer, Center of Biological Sciences, State University of Londrina, Londrina, Brazil; ^2^Laboratory of Immunophysiology of Reproduction, Institute of Biomedical Sciences, Federal University of Uberlândia, Uberlândia, Brazil; ^3^Laboratory of Immunoparasitology, Institute of Biomedical Sciences, Federal University of Uberlândia, Uberlândia, Brazil

**Keywords:** *Toxoplasma gondii*, trophoblast, placenta, nanoparticles treatment, congenital toxoplasmosis

## Abstract

The combination of sulfadiazine and pyrimethamine plus folinic acid is the conventional treatment for congenital toxoplasmosis. However, this classical treatment presents teratogenic effects and bone marrow suppression. In this sense, new therapeutic strategies are necessary to reduce these effects and improve the control of infection. In this context, biogenic silver nanoparticles (AgNp-Bio) appear as a promising alternative since they have antimicrobial, antiviral, and antiparasitic activity. The purpose of this study to investigate the action of AgNp-Bio in BeWo cells, HTR-8/SVneo cells and villous explants and its effects against *Toxoplasma gondii* infection. Both cells and villous explants were treated with different concentrations of AgNp-Bio or combination of sulfadiazine + pyrimethamine (SDZ + PYZ) in order to verify the viability. After, cells and villi were infected and treated with AgNp-Bio or SDZ + PYZ in different concentrations to ascertain the parasite proliferation and cytokine production profile. AgNp-Bio treatment did not reduce the cell viability and villous explants. Significant reduction was observed in parasite replication in both cells and villous explants treated with silver nanoparticles and classical treatment. The AgNp-Bio treatment increased of IL-4 and IL-10 by BeWo cells, while HTR8/SVneo cells produced macrophage migration inhibitory factor (MIF) and IL-4. In the presence of *T. gondii*, the treatment induced high levels of MIF production by BeWo cells and IL-6 by HTR8SV/neo. In villous explants, the AgNp-Bio treatment downregulated production of IL-4, IL-6, and IL-8 after infection. In conclusion, AgNp-Bio can decrease *T. gondii* infection in trophoblast cells and villous explants. Therefore, this treatment demonstrated the ability to reduce the *T. gondii* proliferation with induction of inflammatory mediators in the cells and independent of mediators in chorionic villus which we consider the use of AgNp-Bio promising in the treatment of toxoplasmosis in BeWo and HTR8/SVneo cell models and in chorionic villi.

## Introduction

The intracellular protozoan *Toxoplasma gondii* is the etiologic agent of toxoplasmosis who affects about 30–50% of the world’s population (Montazeri et al., 2017). It is responsible for important clinical conditions in immunocompromised individuals (Atilla et al., 2015; Sutterland et al., 2015; Alday et al., 2017) and congenital toxoplasmosis, mainly in the first gestational months (Kodjikian, 2010). Miscarriage, stillbirth, premature birth, malformations and neurological and/or ocular disorders are examples of symptoms presented in newborns infected by *T. gondii* (Carlier et al., 2012; Li et al., 2014; Oz, 2014), which become the congenital toxoplasmosis is a severe public health problem in many countries, including Brazil (Dubey et al., 2012; Carellos et al., 2014).

*Toxoplasma gondii* infection induces a Th1 immune response with accentuated production of pro-inflammatory cytokines such as IFN-γ and TNF-α (Yarovinsky, 2014; Ching et al., 2016). Antigen presenting cells produce high IL-12 Levels in response to certain antigens released by tachyzoites and stimulate NK cells to produce high levels of IFN-γ, which in turn induces differentiation of CD4^+^ T cells and stimulates macrophages and dendritic cells to release more IL-12 (Yarovinsky, 2014; Hu et al., 2017). Macrophage migration inhibitory factor (MIF), produced by different cell types, is another cytokine with an important role in *T. gondii* infection (Bernhagen et al., 1998). This cytokine participates in dendritic cell maturation and acts in IL-1β, IL-12 and TNF production in the presence of *T. gondii* (Terrazas et al., 2010). To study the congenital toxoplasmosis, various models are used, especially the cell lineage models and placental villi explants. Our previous studies demonstrated that BeWo cells and villous explants infected by *T. gondii* release MIF and IL-6 (Franco et al., 2011; Gomes et al., 2011; Castro et al., 2013; Barbosa et al., 2014, 2015; Abou-kheir et al., 2017; da Silva et al., 2017).

Treatments of congenital toxoplasmosis are scarce at the moment. When the fetal transmission is not confirmed, antibiotic spiramycin is used to prevent vertical transmission (Peyron et al., 2017) since this macrolide drug is not cross-placental (Montoya and Remington, 2008). When fetal infection is confirmed and in most cases symptomatic of toxoplasmosis, the choice of treatment is the combination of pyrimethamine plus sulfadiazine (Dunay et al., 2018). However, these drugs are not well tolerated by the organism, interacting indistinctly with parasite and host biochemical processes (Sepúlveda-arias et al., 2014), presenting adverse effects, such as bone marrow suppression, causing megaloblastic anemia, leukopenia and granulocytopenia (Petersen, 2007). Given this, alternative compounds for toxoplasmosis are increasingly being investigated (Costa et al., 2009; Sanfelice et al., 2017, 2018; Machado et al., 2020).

In this context, the study of nanoparticles (NPs) has been investigated. The main characteristics of NPs are low toxicity, modulation of pharmacokinetics, increased bioavailability, and the possibility of transporting pharmacological components, which also allows the drug to be delivered directly to the specific target (Khalil et al., 2013; Torres-Santiago et al., 2016). Nanoparticles with silver inserts in their structure, called silver nanoparticles (AgNp), are used for health applications because they have antimicrobial and anti-inflammatory activities (Shrivastava et al., 2007; Adair, 2009; Pourali and Yahyaei, 2016; Scandorieiro et al., 2016) described in Gram-negative and Gram-positive bacteria, including multidrug-resistant strains (Shrivastava et al., 2007; Durán et al., 2016; Scandorieiro et al., 2016), protozoa as Leishmania (Allahverdiyev et al., 2011; Fanti et al., 2018; Isaac-Márquez et al., 2018), some virus (Park et al., 2018; Sharma et al., 2019), and filamentous fungi (Sanguiñedo et al., 2018). In addition, AgNp is able to accumulate in tissues, probably reaching cysts of *T. gondii* (Adeyemi and Sulaiman, 2015). Also, AgNp is involved with the production of microbicide components, such as reactive oxygen species (ROS; Butkus et al., 2004; Bhardwaj et al., 2012). Promising activities anti *T. gondii* promoted by metallic nanoparticles are already described (Gaafar et al., 2014; Adeyemi et al., 2017, 2018; Assolini et al., 2017; Machado et al., 2020), but studies regarding the effect of AgNP synthesized biogenically in human cells of trophoblastic origin infected by these protozoa have not yet been described.

Despite the work involving *T. gondii* and alternative treatments in various cell models, there are no descriptions in the literature about the activity of silver biogenic nanoparticle as an alternative toxoplasmosis compound using trophoblast cell models and also with human chorionic villus explants. In this context, our objective was to investigate the anti-*Toxoplasma* and immunological action of the biogenic silver nanoparticle (AgNp-Bio) against *T. gondii* infection in trophoblastic cells and human chorionic villi.

## Materials and Methods

### Cell Lines

Human trophoblast BeWo cells were commercially acquired from American Type Culture Collection (ATCC, Manassas, VA, United States). The human extravillous trophoblast cells (HTR-8/SVneo), originally generated from villous explants an early pregnancy, was gently provided by Dr. Estela Bevilacqua (University of São Paulo, SP, Brazil). BeWo and HTR-8/SVneo cells were maintained in a humidified incubator at 37°C and 5% CO_2_ using culture flasks of 75cm^2^. RPMI 1640 medium (Cultilab, Campinas, SP, Brazil) supplemented with 100U/ml of penicillin (Sigma Chemical Co., St. Louis, MO, United States), 100μg/ml of streptomycin (Sigma), 25mM HEPES and 10% fetal bovine serum (FBS; Cultilab) were used for cell culture maintenance (Barbosa et al., 2008). According to Ethics Committee of the Federal University of Uberlândia, MG, Brazil (Protocol # 13/2012), studies performed with cell lines acquired commercially do not need ethical approval.

### Chorionic Villous Explants

Human placentas (*n* = 6) were acquired from pregnant women after elective cesarean section deliveries (36–40weeks of gestation) at the Clinical Hospital of Federal University of Uberlândia (HCU-UFU), MG, Brazil. Villous explants were only collected from the placenta of patients with no evidence of infectious disease and other pathologies (hypertension, pre-eclampsia, diabetes, cardiac disease, infectious diseases such as toxoplasmosis with negative serology for anti-*T. gondii* IgG/IgM antibodies, and other manifestations) which could interfere with the results of this study. All women included in this work agreed and provided written informed consent. The collection of the placental tissue was authorized (Protocol # 2.360.812) in accordance with Ethics Committee of the UFU. The villous explants were manipulated immediately after delivery in sterile conditions as follow: Samples were washed with sterile phosphate-buffered saline (PBS) and the dissection was performed using a stereomicroscope to eliminate endometrial tissue and fetal membranes up to 1h after collection. Villi with five or seven free tips and volume around 10mm^3^ were collected according to da Silva et al. (2017) and placed in 96-well plates (one per well) with 200μl RPMI 1640 medium supplemented with 10% FBS, penicillin and streptomycin. The plates were kept at 37°C and 5% CO_2_ for next experiments (Castro-Filice et al., 2014).

### Parasites

*Toxoplasma gondii* tachyzoites (virulent RH strain, 2F1 clone) constitutively expressing the β-galactosidase gene were kindly provided by Dr. Vern Carruthers, Medicine School of Michigan University (United States). Briefly, tachyzoites were maintained by serial passages in BeWo cells cultured in RPMI 1,640 medium containing 2% FBS, 100U/ml penicillin, and 100μg/ml streptomycin at 37°C and 5% CO_2_ (Angeloni et al., 2013).

### Biogenic Silver Nanoparticles

The AgNp-Bio were provided by Prof. Dr. Gerson Nakazato from Laboratory of Basic and Applied Bacteriology of the State University of Londrina, Paraná, Brazil, and prepared according to Durán et al. (2005). Briefly, the AgNp-Bio were obtained after reducing the silver nitrate using strain 551 of *Fusarium oxysporum* from the culture collection of the Molecular Genetics Laboratory of ESALQ-USP (Piracicaba, São Paulo, Brazil). *F. oxysporum* was cultivated for 7days in media containing 0.5% (w/v) yeast extract (Neogen), 2% (w/v) malt extract (Neogen), 2% (w/v) agar (Neogen), and distilled water at 28°C. After the growth, the fungal biomass was added to distilled water at 0.1g/ml and incubated at 28°C for 72h. The solution components were filtrated and AgNO3 (Nuclear) at 1mM was added to fungal-free solution. The system was incubated for several hours at 28°C in the absence of light. Constantly, aliquots were removed from solution system and measured the absorptions using an ultraviolet-visible spectrophotometry (VarianCary50Probe); the peak at 440nm corresponded to the surface Plasmon resonance of AgNp. Particle size was measured through a nanoparticle tracking analysis (NTA) of 1mM AgNp-Bio solution diluted in ultrapure water (1:600) using NanoSight LM10 system (Malvern Instruments Ltd., United Kingdom). Machado et al. (2020) executed the analyses using default settings according to the manufacturer’s protocol (Nanosight Software version 3.1).

### Viability Assay

#### Cells

To evaluate cell lines viability in the presence of AgNp-Bio to establish the non-toxic concentration, the tetrazolium salt colorimetric (MTT) assay was performed according to Mosmann (1983). For this purpose, BeWo and HTR-8/SVneo cells were seeded in 96-well plates at 3 × 10^4^cells/well/200μl and incubated for 24h in RPMI 1640 medium with 10% FBS at 37°C and 5% CO_2_. After this period, the cells were treated with AgNp-Bio (1.562, 3.125, 6.25, 12.5, 25, 50, and 100μM/ml) for 24h. To determine the cell viability for the association of sulfadiazine plus pyrimethamine (SDZ + PYR) the concentrations used were 1.56/0.0624, 3.125/0.125, 6.25/0.250, 12.5/0.500, 25/1, 50/2, 100/4, and 200/8μg/ml for BeWo cells and 25/1, 50/2, 100/4, 200/8, 400/16, 500/20, 600/24, 800/32, and 1000/40μg/ml for HTR-8/SVneo cells. Cells maintained with only RPMI were used as a negative control. After of treatment and removal of supernatants, the cells were incubated with 10μl of MTT reagent plus 90μl medium with 10% FBS for 3h, follow by addition of the 10% sodium dodecyl sulfate (SDS, Sigma) and 50% N,N-dimethyl formamide (Sigma) in the plates. Formazan crystals created were solubilized for 30min (Mosmann, 1983) and after the optical densities were measured at 570nm absorbance in a plate reader (Titertek Multiskan Plus, Flow Laboratories, McLean, VA, United States). The cellular viability was expressed by the percentage of viable cells (cellular viability %) in comparison to control cells (treated with complete medium, considered 100% of cellular viability). Three independents experiments were performed in nine replicates (da Silva et al., 2017).

#### Villous Explants

To evaluate the toxicity of AgNp-Bio in villous explants, lactate dehydrogenase (LDH) assay was performed (Castro-Filice et al., 2014). After collection and culture as mentioned before, villi were treated with AgNp-Bio (25, 50, 200, and 800μM/ml) or a combination of 150μg/ml sulfadiazine plus 200μg/ml pyrimethamine according to Castro-Filice et al. (2014). The SDZ + PYR combination was used for comparison with AgNp-Bio treatment, and the concentration was chosen based on no-toxicity in human villous explants observed in a previously study (da Silva et al., 2017). The villous explants were treated with different drugs concentrations in a period of 24h in RPMI 1640 medium with 10% FBS (complete medium). Villous explants maintained with only RPMI were used as a negative control (100% of viability). Following 24h of incubation, supernatants were collected for measurements of LDH levels to demonstrate the expression of toxicities of drug concentrations, as suggesting by the manufacturer’s instructions (LDH Liquiform, Labtes Diagnostica S.A., Lagoa Santa, MG, Brazil). This method is based on the consumption and decrease of the absorption of NADH at 340nm, which is measured in a DU-70 spectrophotometer (Beckman, Brea, CA, United States) for 2min at 37°C. Data were shown in U/L of LDH enzymatic activity. In parallel, the villi were collected for morphological analyses by hematoxylin and eosin staining in order to verify the integrity after treatments. Three placentas were used, and three independent experiments were performed in five replicates.

### Morphological Analysis

To evaluate the morphological aspects, chorionic villi were submitted to fixation step (10% buffered formalin), and dehydration step (in increasing alcohol concentrations) and were embedded in paraffin. Then, 4μm sections were made using a microtome, placed on glass slides and stained with hematoxylin and eosin. The morphological analyses were performed using a light microscope (BX40 Olympus, Tokyo, Japan; Castro-Filice et al., 2014). Morphological aspects of the syncytiotrophoblast and cytotrophoblast cells, as well as the mesenchyme were analyzed. Three placentas were used, and three independent experiments were performed in five replicates.

### Cells Infection and Intracellular Replication of Parasite

To analyze the effect of AgNp-Bio against *T. gondii* proliferation in BeWo and HTR8/SVneo cells, we performed the proliferation assay according to Castro et al. (2013). Briefly, BeWo cells (3 × 10^4^ cells/200μl/well) and HTR8/ SVneo (1 × 10^4^ cells/200μl /well) were seeded in 96 well plates at 37°C at 5% CO_2_ for 24h. Subsequently, these cells were infected with *T. gondii* tachyzoites of the RH strain (2F1; 3 parasites: 1 cell). After 3h, the treatment was included, with the different drugs concentrations (AgNp-Bio: 3.125, 6.25, 12.5, 50, and 100μM/ml; SDZ + PYR: 100μg/ml/4μg/ml). Cells maintained with only RPMI were used as a negative control (medium). After 24h, plates were centrifuged (400 × *g* for 10min) and the supernatants were removed. Parasites not internalized in the cells were removed by PBS lavage containing 1mM CaCl2 and 1mM MgCl2. Then, 100μl lysis buffer (100mM HEPES, pH 8.0, 1mM MgSO 4, 0.1% Triton X-100, 5mM dithiothreitol), 160μl assay buffer (100mM phosphate buffer, pH 7.3, 102mM β-mercaptoethanol, 9mM MgCl_2_) and (40μl of the substrate CPRG; Roche) and incubated for 30min at room temperature in the dark. After, the enzymatic activity of β-galactosidase was measured at 570nm using plate reader (Titertek Multiskan Plus, Flow Laboratories, McLean, United States). Standard curve analyzes and tachyzoite proliferation index were performed according to Barbosa et al. (2015). Three independent experiments were performed in nine replicates. The supernatant from the cells was collected and stored at −80°C for cytokines detection.

### Villous Explants Infection and Intracellular Replication of Parasite

Villous explants cultured in complete medium were infected with *T. gondii* tachyzoites of the RH strain (2F1; 1 × 10^6^ parasites per well). After 24h, the villi were washed with complete medium to remove non-adherent parasites and treated for an additional 24h with AgNp-Bio (25, 50, 200, and 800μM/ml) or a combination of 150μg/ml sulfadiazine plus 200μg/ml pyrimethamine. Villous explants maintained with only RPMI were used as a negative control. After, supernatants were collected and stored at −80°C for cytokine detection. Villous explants were collected for morphological analysis or *T. gondii* proliferation by the β-galactosidase colorimetric assay (Barbosa et al., 2014, 2015; da Silva et al., 2017) or immunohistochemistry assay.

To quantify the intracellular parasite proliferation in villi, the protein content was determined as follows: addition of 150μl RIPA buffer [50mM Tris-HCl, 150mM NaCl, 1% Triton X-100, 1% (w/v) sodium deoxycholate, and 0.1% (w/v) sodium dodecyl sulfate (SDS), pH 7.50] supplemented with protease inhibitor cocktail (Complete, Roche Diagnostic, Mannheim, Germany) to each villous and homogenizing the samples on ice for protein extraction. The homogenate was centrifuged (21,000 × *g* for 15min at 4°C) and the supernatant was collected. The total protein (μg/ml) was measured by Bradford assay (Bradford, 1976). Aliquots of 20μl of each sample were used to determine *T. gondii* intracellular proliferation by β-galactosidase assay, as described above (da Silva et al., 2017). Next, the number of tachyzoites was normalized according to the protein concentration of each villous, showing the number of tachyzoites per μg of tissue. In parallel, a standard curve was constructed by serial dilution, in duplicate of *T. gondii* tachyzoites and the data were shown as *T. gondii* proliferation index (number of tachyzoites) in comparison to a standard curve of free tachyzoites (1 × 10^6^–15.62 × 10^3^; Barbosa et al., 2015). Three samples of placenta were collected and three independent experiments were performed in nine replicates.

### Immunohistochemistry Assay

For immunohistochemistry assay, chorionic villi were submitted to the fixation step (in formalin 10%) and the dehydration step (in increasing alcohol concentrations) and embedded in paraffin, in parallel of the morphological analysis. Then, sections with 4μm were confectioned in microtome, placed on glass slides and subjected to immunohistochemical analysis (Gomes et al., 2011; Castro-Filice et al., 2014). Briefly, sections were covered with citric acid pH 6.0 for 5min in a microwave for antigenic retrieval. The sections were incubated with 5% acetic acid solution for 8min at room temperature to block endogenous phosphatase activity and reduce the nonspecific binding. After, the sections were incubated with 2.5% goat serum for 45min at 37°C. The sections were incubated overnight at 4°C with *Calomys callosus* serum infected with *T. gondii* (1:100). On the following day, biotinylated goat-anti mouse IgG (1:600, Jackson Immuno Research Laboratories, West Grove, PA, United States) secondary antibody was added to the section for 1h at 37°C. The reaction was developed with fast red naphthol (Sigma), the tissue counterstained with Harris’s hematoxylin and analyzed under a light microscope (BX40, Olympus, Tokyo, Japan; Gomes et al., 2011; Castro-Filice et al., 2014).

### Determination of Cytokine Levels

The cytokines IL-4, IL-6, IL-8, IL-10, MIF, and TNF-α were assessed in BeWo or HTR-8/SVneo cells supernatants. The molecules were measured by sandwich enzyme-linked immunosorbent assay (ELISA: BD Biosciences, San Diego, CA, United States; or R&D Systems, Minneapolis, MN, United States) and data were expressed in pg/ml. Besides, MIF, IL-4, IL-8 e IL-6 cytokines were measured in supernatants of villous explants, according with manufacturer’s instructions. For villous explants, the data were normalized according to the protein concentration of each villous as described above and obtained by the ratio between concentration of cytokines in pg/ml and concentration of total protein from Bradford assay in μg/ml, resulting in pg/μg of tissue. The limits of detection of each cytokine were determined from standard curves as follow: IL-6 = 4.7, IL-8 = 0.8, TNF-α = 7.8, MIF = 7.8, and IL-10 = 7.8 (all in pg/ml).

### Parasite Treatment and Viability

To evaluate the influence of treatment in the parasite viability, tachyzoites (2F1 clone; 1 × 10^6^) were added in microtubes and treated with different concentrations of AgNp-Bio in a period of 3h in RPMI 1640 medium with 5% FBS at 37°C and 5% CO_2_. In the next step, the parasites were stained with Trypan blue, enabling the identification of viable tachyzoites from observation of clear cytoplasm and negative trypan blue staining, and unviable parasites from observation of dark cytoplasm and positive trypan blue staining. The parasites were counted under an optical microscope (Castanheira et al., 2015). As control, tachyzoites were treated with only medium.

### Statistical Analysis

Differences between groups were assessed by One-Way ANOVA and Bonferroni multiple comparison *post hoc* test, or Kruskall Wallis with Dunn’s multiple comparison *post hoc* test, when appropriate. Data were expressed as mean ± standard error of mean (SEM) of the experimental groups. Statistical differences were considered significant when *p* < 0.05. The GraphPad Prisma Software 5.0 (GraphPad Software, Inc., San Diego, CA, United States) was used to analysis.

## Results

### Cells and Villous Explants Maintain Viability After Treatment With AgNp-Bio

**Figure 1:**
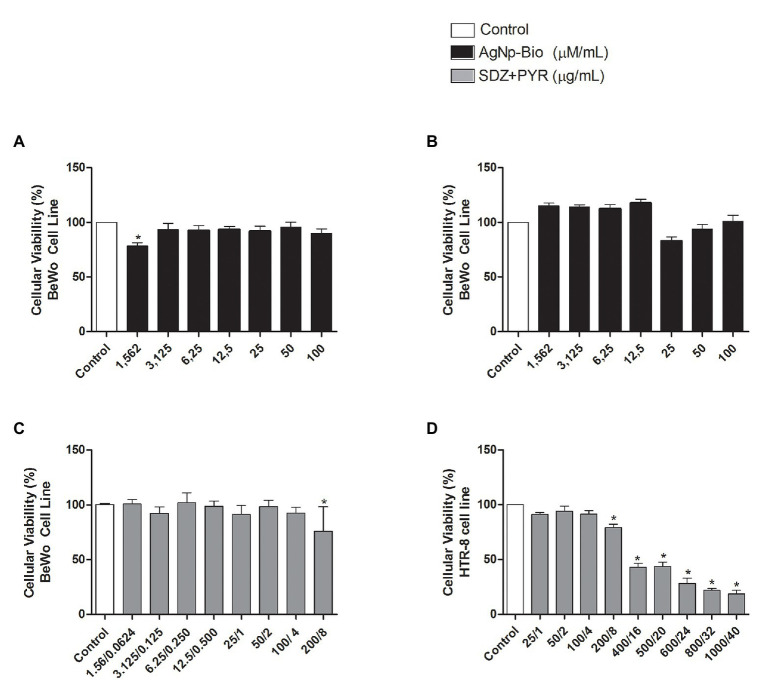
Cellular viability after treatment with different nanoparticles concentrations. **(A)** BeWo cells were cultured in 96-well plates (3 × 10^4^ cells/well/200μl) for 24h and treated with biogenic silver nanoparticles (AgNp-Bio) concentration (μM/ml) or combination of sulfadiazine/ pyrimethamine (SDZ + PYR) or complete medium (negative control). **(B)** HTR8/SV neo cells were cultured in 96-well plates (3 × 10^4^ cells/well/200μl) for 24h and treated or untreated with nanoparticle in different concentrations (μM/ml) or complete medium (control). **(C)** Combination of SDZ + PYR in BeWo cells (*p* < 0.05). **(D)** Combination of SDZ + PYR in HTR-8 cells (*p* < 0.05). Data were shown as mean ± SEM from three independents experiments with nine replicates. Significant differences in relation to untreated cells (control; ^*^p < 0.05). Differences between groups were analyzed by One-Way ANOVA with the Dunnett’s multiple comparison *post hoc* test.

In order to verify the possible cytotoxicity of AgNp-Bio in BeWo and HTR-8/SVneo cells the MTT assay was performed (Figure 1). For BeWo cells, the viability reduced only at the smallest concentration (1.56μM/ml) in relation to non-treated controls (*p* < 0.05; Figure 1A). No change in the viability was observed for HTR-8/SVneo cell treated with AgNp-Bio (Figure 1B). In addition, BeWo cell did not show significant difference in viability when treated with combination of sulfadiazine plus pyrimethamine (SDZ + PYZ) except at 200 + 8μg/ml concentration (Figure 1C). HTR8/SVneo cells treatment with SDZ + PYZ induced reduction in the cellular viability at, 200 + 8; 400/16; 500 + 20; 600 + 24; 800 + 32, and 1000 + 40μg/ml when compared to untreated cells (Figure 1D; *p* < 0.05). Based on these data, the drug concentrations selected to carry out further experiments with cells were 3,125; 6,25; 12,5; 50; 100μM/ml for AgNp-Bio and 25/1; 50/2; 100/4μg/ml for SDZ + PYZ.

**Figure 2:**
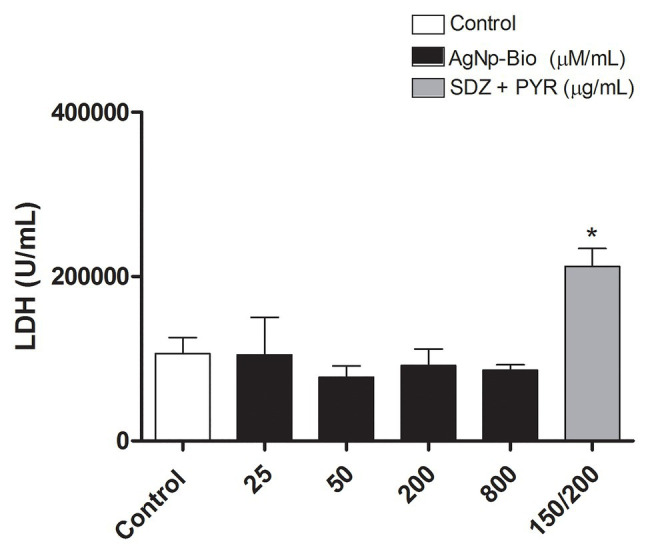
Analysis of toxicity in human villous explants after treatment with different nanoparticles concentration. Villous explants were cultured in a period of 24h in 96-well plates and treated with AgNp-Bio concentration (μM/ml) or combination of sulfadiazine/ pyrimethamine (SDZ + PYR) or complete medium (negative control). After, supernatants were collected and lactate dehydrogenase (LDH) activity was measured using the LDH Liquiform Kit. Three placentas were used, and three independent experiments were performed in five replicates. Data were expressed as mean ± SEM from three independent experiments in five replicates. Comparison in relation to negative control (medium; Control -; ^*^p < 0.05). Differences between groups were analyzed by One-Way ANOVA with the Dunnett’s multiple comparison *post hoc* test.

**Figure 3:**
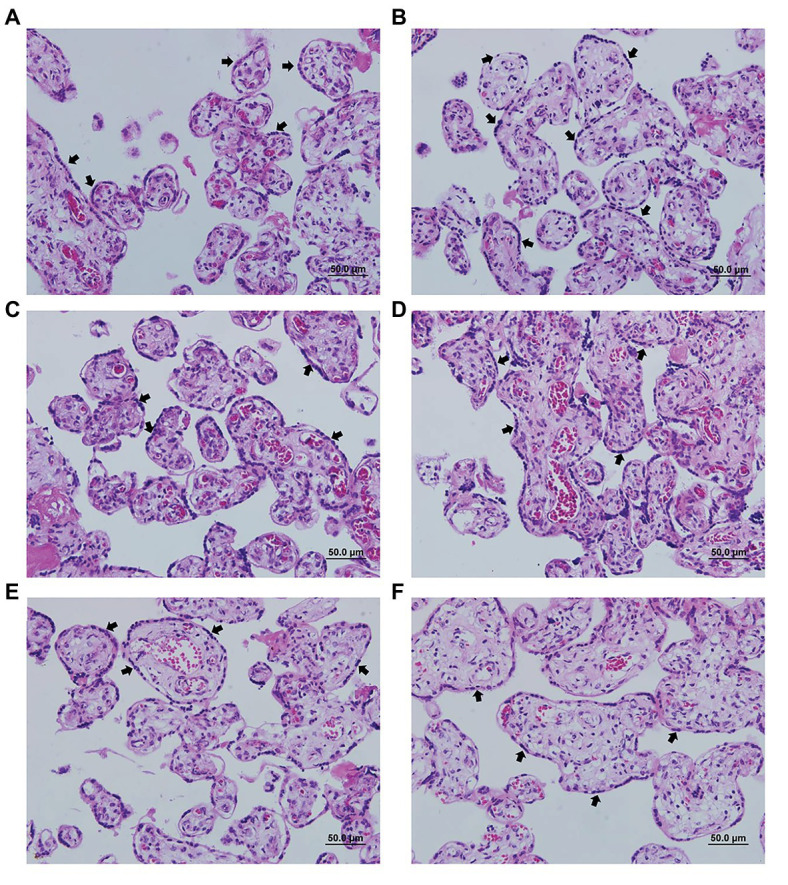
Representative photomicrograph of villi treated or not with different concentrations of nanoparticles. **(A)** negative control (untreated); **(B)** 25μM/ml; **(C)** 50μM/ml; **(D)** 200μM/ml; **(E)** 800μM/ml; and **(F)** positive control treated with sulfadiazine/ pyrimethamine (SDZ + PYR). The arrows indicate the cell layer of the cytotrophoblast and the integrity of the tissue. Hematoxylin and eosin staining.

In order to evaluate the possible cytotoxicity of AgNp-Bio or SDZ + PYZ in villous explants the LDH assay was performed (Figure 2). Also, the morphology was analyzed Figure 3. No significant difference in tissue viability was observed in villous explants, regardless of treatment, when compared to the negative control (Control-; Figure 2). The treatment with either drug did not trigger morphological change in villous explants (Figures 3A–F). Syncytiotrophoblasts covering the chorionic villous were observed, but there were no morphological changes in the cytotrophoblasts and mesenchymal tissue (Figures 3A–F).

### AgNp-Bio Reduce *T. gondii* Intracellular Proliferation in BeWo and HTR8/SVneo Cells

**Figure 4:**
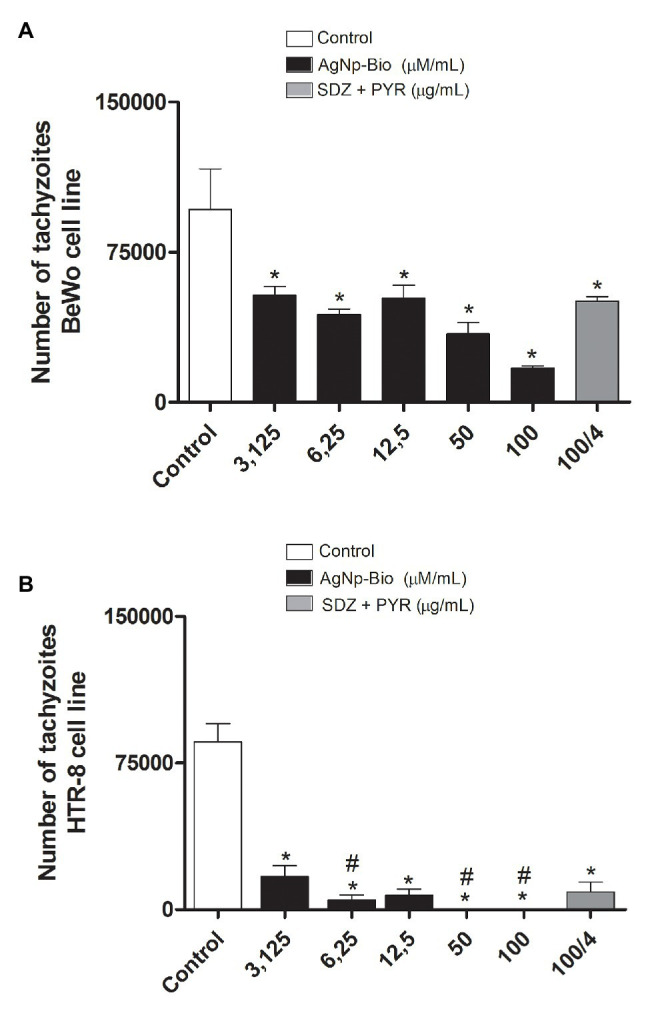
*Toxoplasma gondii* proliferation (2F1 clone) in BeWo **(A)** and HTR8 **(B)** treated with different drugs. After culturing and infecting the cells with *T. gondii* (RH-2F1 clone) for 24h, treatment with the nanoparticles and the combination of sulfadiazine/pyrimethamine (SDZ + PYR) was performed for later β-galactosidase colorimetric assay. Data were shown as mean ± SEM of *T. gondii* proliferation from three independents experiments in nine replicates. Significant differences in relation to BeWo, HTR8 cells infected and untreated (control; ^*^p < 0.05; **A,B**) and nanoparticles and SDZ + PYR (^≠^*p* < 0.05; **A,B**). Differences between groups were analyzed by One-Way ANOVA with the Bonferroni multiple comparison *post hoc* test.

BeWo and HTR8/SVneo cells were treated for 24h with drugs for the assay β-galactosidase. The AgNp-Bio treatment, independently of concentration significantly reduced the intracellular proliferation of *T. gondii* in both cell lines when compared to control (Figures 4A,B). Also, SDZ + PYZ treatment was able to reduce parasite intracellular proliferation (Figures 4A,B; *p* < 0.05).

In HTR8 / SV neo cells, when we compare the different concentrations of AgNpBio with the concentrations of SDZ + PYZ, we observed that the treatment with AgNpBio showed significantly lower levels of *T. gondii* proliferation at concentrations 6.25; 50, and 100μM/ml than in treatments with the association of SDZ + PYZ (100/4μg/ml; *p* < 0.05; Figure 4B).

### AgNp-Bio Reduce Tissue Parasitism in Villous Explants

**Figure 5:**
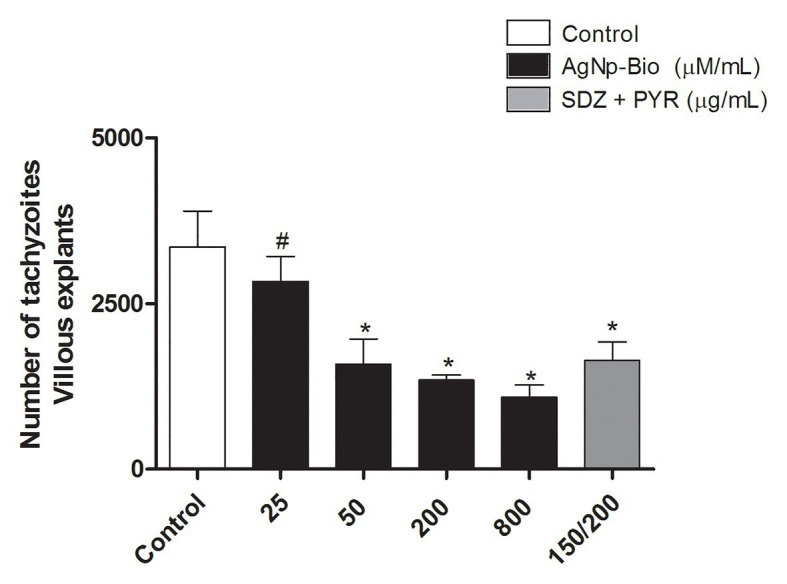
*Toxoplasma gondii* proliferation (2F1 clone) in villi treated with different drugs. Villi were collected and cultured for 24h, infected with *T. gondii* (RH-2F1 clone) for 24h, and treated or untreated with nanoparticles in different concentration and sulfadiazine/ pyrimethamine (SDZ + PYR). Next, were treated for β-galactosidase colorimetric assay. Data were shown as mean ± SEM of *T. gondii* proliferation from three independents experiments in nine replicates. Significant differences in relation infected and untreated (control; ^*^p < 0.05) and nanoparticles and SDZ + PYR (^≠^*p* < 0.05). Differences between groups were analyzed by One-Way ANOVA with the Bonferroni multiple comparison *post hoc* test.

No significant difference in intracellular proliferation of *T. gondii* was observed when villi were treated with AgNp-Bio in concentrations of 25μM/ml compared to control, whereas higher concentrations (50, 200, and 800μM/ml) exhibited significant reduction in *T. gondii* intracellular proliferation. Also, SDZ + PYZ decreased the tissue parasitism when compared to control (Figure 5; *p* < 0.05).

**Figure 6:**
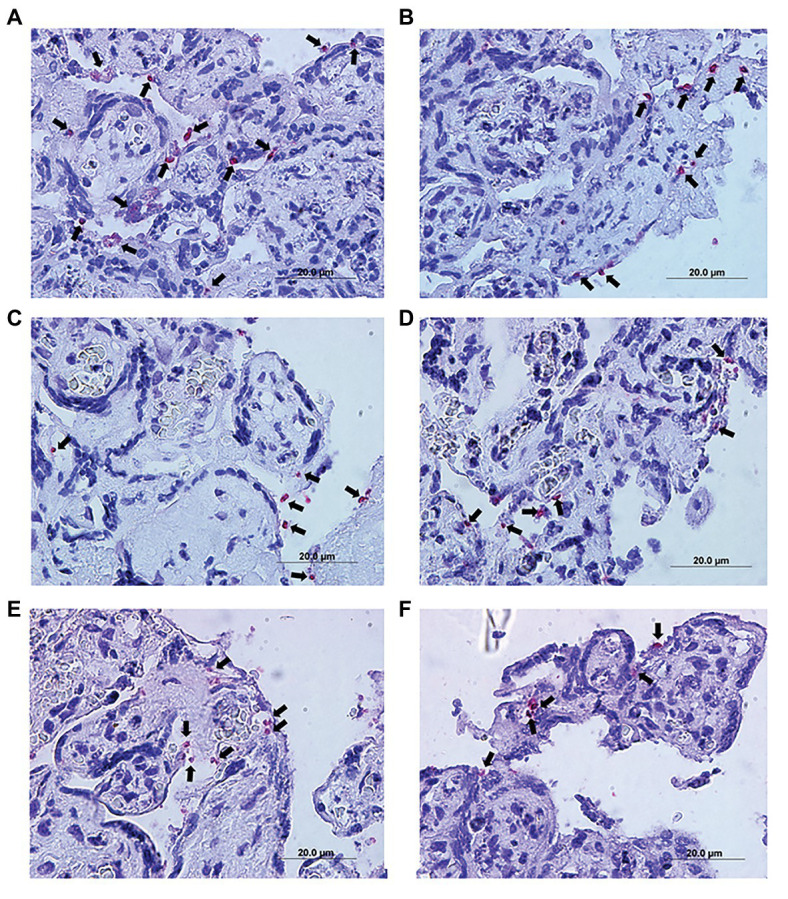
Representative photomicrograph of infected villous explants and treated with different AgNp-Bio concentration or SDZ + PYR. **(A)** Control; **(B)** 25μM/ml; **(C)** 50μM/ml; **(D)** 200μM/ml; **(E)** 800μM/ml; and **(F)** SDZ + PYR. The arrows indicate *T. gondii* tachyzoites immunolocated by immunophosphatase staining and counterstained with hematoxylin.

Immunohistochemical detected the presence of *T. gondii* in villous explants (Figures 6A–F). A smaller number of tachyzoites (arrows) were observed in villi treated with higher AgNp-Bio concentrations or SDZ + PYZ compared to control (Figures 6A–F).

Also, it was possible to observe no change in the tissue structure, presenting normal morphology of the syncytiotrophoblast cells and mesenchyme when compared to control (Figures 6A–F).

### AgNp-Bio Induces Production of IL-4 and IL-10 in BeWo Cells

**Figure 7:**
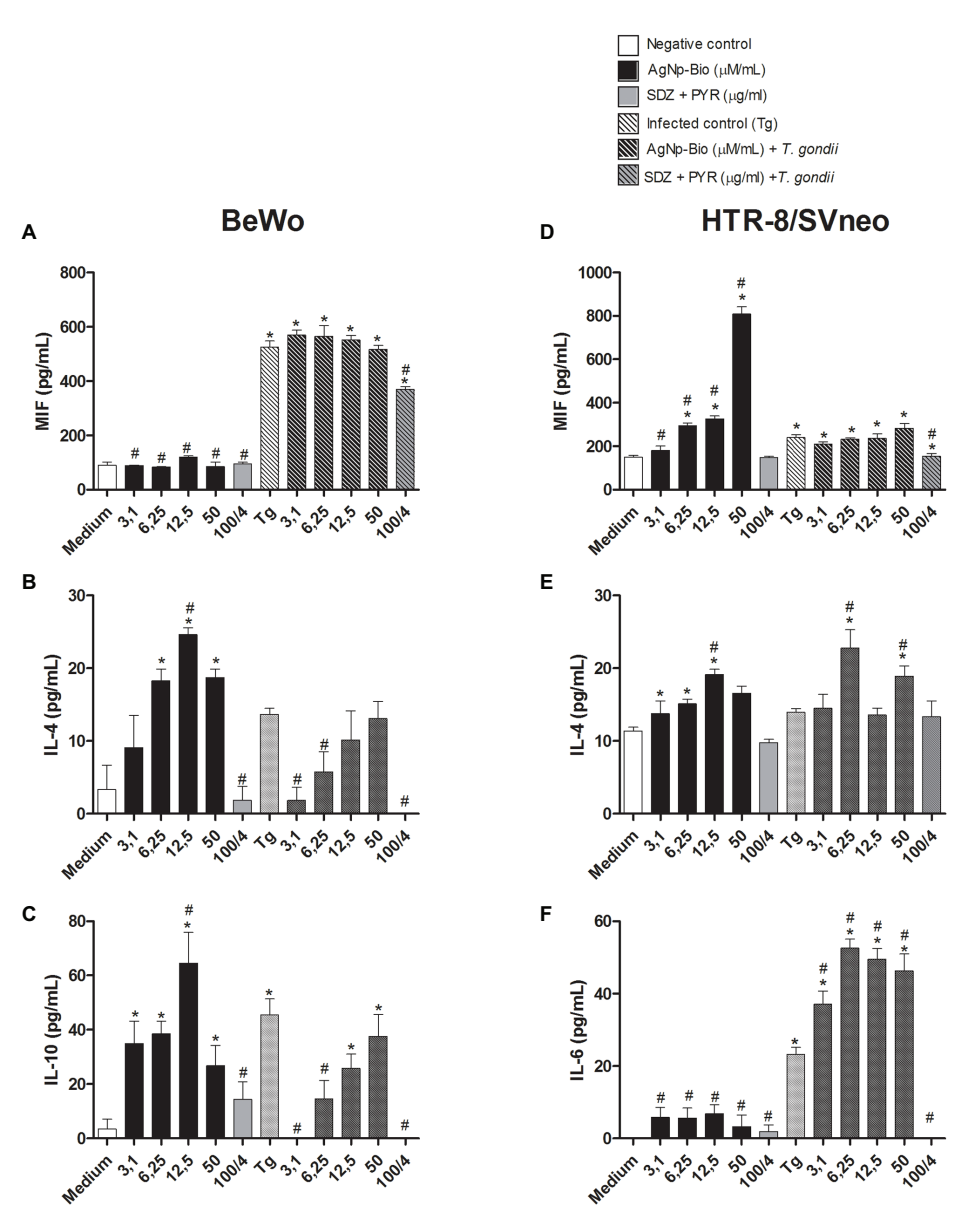
Citokines production in BeWo and HTR-8 cells treated with different drugs and infected or not with *Toxoplasma gondii* (RH2F1 clone). BeWo and HTR-8/SVneo cells were cultured in plates for 24h, infected or not with *T. gondii* (RH2F1 clone) for 3h. After, cells were treated or untreated with different concentration of AgNp-Bio or combination of sulfadiazine/pyrimethamine (SDZ + PYR). Cell-free supernatants were collected after 24h of treatment and the cytokines production was measured by sandwich ELISA. Production of macrophage migration inhibitory factor (MIF; **A**), IL-4 **(B)** and IL-10 **(C)** by BeWo and production of MIF **(D)**, IL-4 **(E)**, IL-6 **(F)** by HTR-8/SVneo. Data were expressed in pg/ml and shown as mean ± SEM from three independents experiments in nine replicates. Comparison in relation to control (control; ^*^p < 0.05). Comparison in relation to Tg (^#^*p* < 0.05). Differences between groups were analyzed by One-Way ANOVA with the Bonferroni multiple comparison *post hoc* test.

When the MIF cytokine was analyzed, it was observed that uninfected BeWo cells treated with AgNp-Bio or SDZ + PYZ did not change the MIF production in relation to medium (Figure 7A). On the other hand, infected cells (Tg) or infected cells treated with AgNp-Bio or SDZ + PYZ significantly increased MIF release in comparison to medium (Figure 7A). In addition, uninfected BeWo cells treated with AgNp-Bio or SDZ + PYZ and infected BeWo cells treated with SDZ + PYZ produced lower MIF levels in comparison to Tg (*p* < 0.05; Figure 7A). No statistical difference in MIF levels was observed when infected BeWo cells treated with AgNp-Bio were compared with Tg (Figure 7A).

Uninfected BeWo cells treated with AgNp-Bio at concentrations of 6.25, 12.5, or 50μM/ml increased IL-4 release in comparison to uninfected/untreated cells (medium; Figure 7B). No statistical difference in IL-4 production was observed when uninfected BeWo cells treated with AgNp-Bio at concentrations of 3.1μM/ml or treated with SDZ + PYZ were compared with medium (Figure 7B). The same result was observed in infected BeWo cells, regardless of treatment (Figure 7B). Moreover, uninfected BeWo cells treated with AgNp-Bio at concentration of 12.5μM/ml produced higher IL-4 Levels in comparison to infected/untreated (Tg; *p* < 0.05; Figure 7B).

Uninfected BeWo cells treated with SDZ + PYZ or infected cells treated with AgNp-Bio at concentrations of 3.1, 6.25μM/ml or SDZ + PYZ produced lower IL-4 Levels compared to Tg (*p* < 0.05; Figure 7B). No statistical difference in IL-4 production was observed by infected BeWo cells treated with AgNp-Bio at concentrations of 12.5 and 50μM/ml compared with Tg (Figure 7B).

The AgNp-Bio treatment, independently of concentrations, increased IL-10 release in comparison to medium (Figure 7C). No statistical difference in IL-10 production was observed when uninfected BeWo cells treated with SDZ + PYZ were compared with medium (Figure 7C). Infected cells (Tg) and infected cells treated with 12.5 or 50μM/ml produced higher IL-10 Levels in comparison to medium (Figure 7C). Uninfected BeWo cells treated with AgNp-Bio at concentrations of 12.5μM/ml increased IL-10 release in comparison to Tg (Figure 7C). No statistical difference in IL-10 Levels was observed when uninfected BeWo cells treated with others AgNp-Bio concentrations compared with Tg (Figure 7C). On the other hand, infected cells treated with AgNp-Bio at concentrations of 3.1and 6.25μM/ml or treated with SDZ + PYZ decrease IL-10 production in comparison to Tg (Figure 7C).

IL-6 cytokine was detected just in infected cells and no significant difference was observed between experimental conditions. In addition, IL-8 and TNF were not detected in BeWo cells in any experimental conditions (data not shown).

### AgNp-Bio Induces Production of MIF and IL-4 in HTR8/SVneo, While Il-6 Is Upregulated by *T. gondii* and AgNp-Bio

Uninfected HTR8/SVneo cells treated with AgNp-Bio at concentrations of 6.25, 12.5, and 50μM/ml increased MIF release in comparison to medium (Figure 7D). No statistical difference in MIF production was observed when uninfected HTR8/SVneo cells treated with AgNp-Bio at concentrations of 3.1μM/ml or treated with SDZ + PYZ were compared with medium (Figure 7D).

Uninfected cells treated AgNp-Bio, independently of concentrations, increased MIF release in comparison to Tg (Figure 7D).

The infected HTR8/SVneo cells or infected cells treated with AgNp-Bio, regardless of concentrations, increased MIF release in comparison to medium (Figure 7D). On the other hand, infected HTR8/SVneo cells treated with SDZ + PYZ produced lower levels of MIF compared with medium (Figure 7D). No statistical difference in MIF levels was observed when infected HTR8/SVneo cells treated with AgNp-Bio were compared with Tg (Figure 7D). Infected HTR8/SVneo cells treated with SDZ + PYZ produced lower levels of MIF compared with medium (Figure 7D).

Uninfected HTR8/SVneo treated with AgNp-Bio at concentrations of 3.1, 6.25, and 12.5μM/ml increased IL-4 release in comparison to medium (Figure 7E). No statistical difference in IL-4 Levels was observed when uninfected BeWo cells treated with AgNp-Bio at concentration of 50μM/ml or SDZ + PYZ were compared with medium (Figure 7E). Infected HTR8/SVneo treated with AgNp-Bio at concentrations of 6.25 and 50μM/ml increased IL-4 release in comparison to medium (Figure 7E). No statistical difference in IL-4 Levels was observed when infected HTR8/SVneo cells or infected HTR8/SVneo cells treated with AgNp-Bio at concentration of 3.1, 12.5μM/ml or SDZ + PYZ were compared with medium (Figure 7E). When compared with Tg, just uninfected HTR8/SVneo cells treated with AgNp-Bio at concentrations of 12.5μM/ml increased IL-4 release. At the same way, just infected HTR8/SVneo cells treated with AgNp-Bio at concentration of 6.5 or 50μM/ml increased IL-4 release (Figure 7E).

When the IL-6 cytokine was analyzed, it was observed that uninfected HTR8/SVneo cells treated with AgNp-Bio or SDZ + PYZ did not change the IL-6 in relation to medium (Figure 7F). However, infected HTR8/SVneo cells, treated or not with AgNp-Bio significantly increased IL-6 release in comparison to medium (Figure 7F). No statistical difference in IL-6 Levels was observed when infected HTR8/SVneo cells treated with SDZ + PYZ were compared with medium (Figure 7F). In addition, uninfected HTR8/SVneo cells treated with AgNp-Bio or SDZ + PYZ produced lower IL-6 Levels in comparison to Tg (*p* < 0.05; Figure 7F). However, infected HTR8/SVneo cells treated with AgNp-Bio or SDZ + PYZ significantly increased IL-6 release in comparison to Tg (Figure 7F).

IL-10 cytokine was detected, but just uninfected cells treated with AgNp-Bio at concentrations of 12.5μM/ml showed increase in IL-10 production compared with medium (data not shown). No significant difference was observed between others experimental conditions. IL-8 and TNF were not detected in HTR8/SVneo cells under any experimental conditions (data not shown).

### AgNp-Bio Downregulated Production of IL-4, IL-6, and IL-8 in Infected Villi

**Figure 8:**
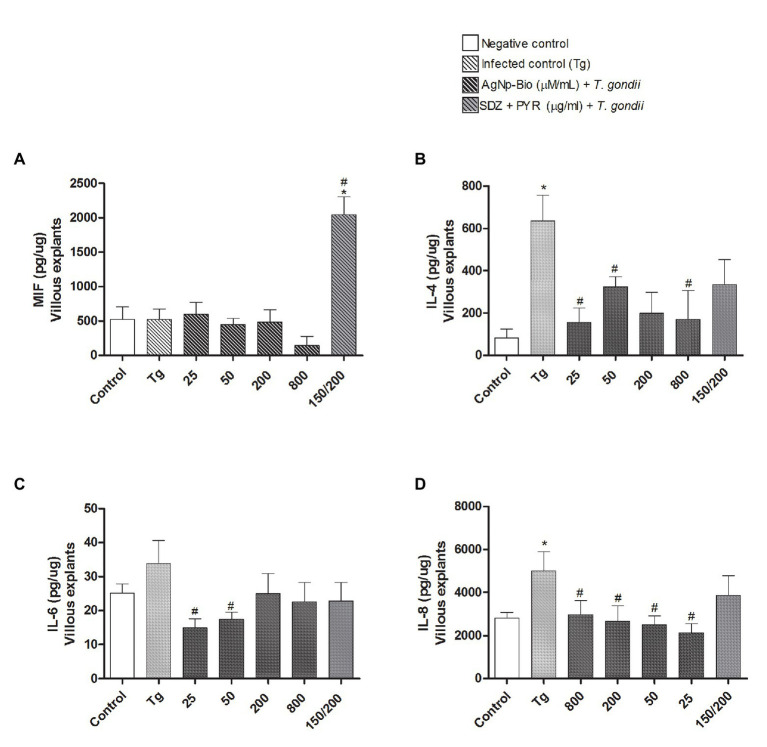
Citokines production by villous explants. Villi were cultured and infected or not with *T. gondii* for 24h. After, villi were treated or untreated with different AgNp-Bio concentration or combination of sulfadiazine/ pyrimethamine (SDZ + PYR). After 24h of treatment, supernatants were collected and the cytokines production was measured by sandwich ELISA. Production of MIF **(A)**, IL-4 **(B)**, IL-6 **(C)** and IL-8 **(D)**. Data were expressed as pg/ng and shown as mean ± SEM from three independents experiments in nine replicates. Comparison in relation to control (control; ^*^p < 0.05). Comparison in relation to Tg (^#^*p* < 0.05). Differences between groups were analyzed by One-Way ANOVA with the Bonferroni multiple comparison *post hoc* test.

After analyzing the *T. gondii* replication in infected villous explants, the profile of cytokines was determined (Figure 8).

When the MIF cytokine was analyzed, it was observed that only infected villous explants treated with SDZ + PYZ showed increase of MIF when compared to medium and Tg (Figure 8A).

When analyzing the cytokines IL-4, IL-6, and IL-8, we did not observe any statistically significant difference between the chorionic villi infected and treated with AgNp-Bio or SDZ + PYZ when compared to the medium (*p* < 0.05; Figures 8B–D respectively).

Infected villous explants treated with AgNp-Bio, independently of concentrations, produced lower IL-4 (except in concentration 200μM/ml) and IL-8 Levels in comparison to Tg (*p* < 0.05; Figures 8B,D). No statistical difference was observed between infected villous explants treated with SDZ + PYZ and Tg for both IL-4, IL-6, and IL-8 cytokines (Figures 8B–D).

No statistical difference in IL-6 Levels was observed when infected villous explants, treated or not were compared with medium (Figure 8C). Infected villous explants treated with AgNp-Bio at concentration of 25 or 50μM/ml produced lower IL-6 Levels in comparison to Tg (*p* < 0.05; Figure 8C). No statistical difference was observed between infected villous explants treated with AgNp-Bio at concentration of 200 or 800μM/ml or SDZ + PYZ and Tg (Figure 8C).

## Discussion

When *T. gondii* infection occurs during pregnancy, vertical transmission can be established (Li et al., 2014), leading possible manifestations in newborns, such as chorioretinitis, intracranial calcifications, hydrocephalus, and neurological disabilities (Maenz et al., 2014). Studies using BeWo, HTR8/SVneo, and chorionic villus explants are very important for clear the interactions of pathogens, drugs, and elements from other fields of medicine, including nanotechnology, in maternal interface (de Araújo et al., 2019).

In this study we used the Biogenic Silver Nanoparticles (AgNp-Bio). The chemical properties of this compound providing the ability to transport pharmacological agents into the target tissue, due to the large surface area and small size structure (Riehemann et al., 2009), leading to a decrease in side effects and greater specificity, when compared to other conventional treatments (Khanna et al., 2015). Furthermore, among the different methods applied to synthetize nanoparticles, the biogenic process has the best performance and stability as well as the lowest toxicity (Gurunathan et al., 2014; Pourali and Yahyaei, 2016; Girilal et al., 2018). Accordingly, previous studies confirm the theory that the biosynthesis of AgNp using bacteria (Das et al., 2014), fungi (Velhal et al., 2016), and plants (Arokiyaraj et al., 2017) have fewer toxic effects (Velhal et al., 2016; Anbazhagan et al., 2017).

In the present study, BeWo or HTR8/SVneo cells treated with AgNp-Bio maintained cellular viability in comparison to untreated cells. Regarding cells, similar data were obtained by Rahul et al. (2015), in turn, found that AgNp associated with pigments obtained of bacteria did not present significant toxicity in breast cancer cells (MCF7), in HeLa cells and peripheral blood mononuclear cells (PBMCs). Already, Machado et al. (2020) revealed dose-dependent cytotoxicity of the AgNp-Bio in a HeLa cell.

At the same way, in the present study, villous explants maintained tissue viability after treatments. The absence of cytotoxicity is relevant, considering that the synchiotrophoblast layer is susceptible to environmental conditions and that its cells can be damaged if its functionality is changed. Therefore the analysis of placental villi explants morphology is extremely important for assessing the tissue viability *in vitro* (Miller et al., 2005). In addition da Silva et al. (2017) showed no toxicity for human villous explants after enrofloxacin, toltrazuril, and azithromycin treatment.

The size of nanoparticles is a crucial factor to toxicity, since smaller nanoparticles present significant toxic effects due to a larger surface area and increased uptake into cells and capacity of translocate across cell layers (Oberdörster et al., 2005). Many studies did not observe toxic effects in different human cells after AgNp incubation with diameters from 6 to 80nm (Hussain et al., 2005; Asharani et al., 2009; Lu et al., 2010; Foldbjerg et al., 2011). The AgNp-Bio used in this study has a mean size of 69.0nm, thus we believe that it to be a factor to justify the low toxicity of this nanomaterial in our experimental models.

The conventional treatment of congenital toxoplasmosis presents a toxic effect for pregnant women (Montoya and Remington, 2008), demonstrated the importance of studies about new therapies to prevent effects triggered by *T. gondii* vertical transmission. In the present study, it was observed that AgNp-Bio treatment reduced *T. gondii* proliferation in BeWo and HTR8/SVneo cells with an inhibition of 82.2 and 100%, respectively. Interestingly, a significant reduction in parasite proliferation in AgNp-Bio treated cells was observed when compared with SDZ + PYZ treatment. It is true to chorionic villus explants and the parasite reduction was dose-dependent. Similar results using HeLa cells were observed by Machado et al. (2020) regarding the reduction in parasite proliferation rates. Besides that, Adeyemi et al. (2017) evaluated the anti-*T. gondii* action of gold, silver and platinum nanoparticles and observed reduction in the parasite invasion and replication. In other study, Gaafar et al. (2014) related that the AgNp had an anti-*Toxoplasma in vivo* effect with lower parasite load in liver and spleen. In the same study, the parasites presented deformations with the formation of irregular grooves and disorder of the conoid and plasmatic membrane. According to Zheng et al. (2008), the AgNp can be stored inside mitochondria, thus hampering the synthesis of adenosine triphosphate (ATP) as well as causing damages to the mitochondrial membrane (Adeyemi et al., 2017). In addition, the AgNp are able to interact with leucine aminopeptidase enzyme (LAP) triggering a rupture of its protein structure and causing a loss of function (Mnkandhla et al., 2018). Consequently, impairment of the parasitic activity of *T. gondii* occurs, whereas LAP has essential roles on physiological processes in these protozoa (Matsui et al., 2006; Jia et al., 2010). Studies with nanoparticles and protozoa, mostly, are related to AgNp; however, investigations with AgNp-Bio and protozoa are scarce (Fanti et al., 2018; Machado et al., 2020).

The placenta model presents the advantage to maintain the *in vivo* architecture of the tissue (Balan et al., 2017; Valero et al., 2018) when compared to primary cellular culture of trophoblasts or co-cultures 3D model (Muoth et al., 2016), which could favor the treatment during pregnancy. In some way, it was demonstrated by Dolati et al. (2016) using polymeric NPs. These authors observed that polymeric NPs release can be more slowly and thus require less frequent administration which could be an advantage in the treatment of pregnant women, targeting both the fetus and the mother. In this sense, NPs compared to conventional treatment used in the clinic, can be considered a good alternative treatment for no presenting cytotoxic results even at higher doses, while classical treatment with pirymethamine induced increased cytotoxicity in low doses.

In the present study, we did not investigate increased doses of sulfadiazine plus pyrimethamine association since they were toxic all of the placental tissue in previous study by our group (Castro-Filice et al., 2014). This observation demonstrated that standard treatment is very toxic to placental tissues, causing damages of the materno-fetal interface in elevated doses and did not guarantee the successful control of *T. gondii* proliferation (da Silva et al., 2017). In contrast, some antibiotics from the fluoroquinolone group are not associated with teratogenic effects to the fetus, which can be a promisor alternative for the treatment (Larsen et al., 2001). Thus, AgNp-Bio is able to decrease *T. gondii* proliferation in BeWo, HTR8SV/neo, and villous explants more efficiently that classical treatment.

Protective immune responses against pathogens during pregnancy are modulated by induction of tolerance against the semi-allogeneic fetus (Montoya and Remington, 2008). Proinflammatory mediators play a central role in trigger acute and chronic inflammatory processes. Acute *T.gondii* infection during gestation can damage the maternal-fetal immunological balance in favor of anti-parasitic pro-inflammatory abortogenic cytokines (Shiono et al., 2007). For these reasons, we decided to verify the cytokine profile when trophoblast cells were treated with AgNp-Bio. When we investigated the AgNp-Bio action in cytokines production by both cell lineages, we observed increase of IL-4 and IL-10 by BeWo, while HTR8/SVneo cells produced MIF and IL-4. Thus, the treatment with nanoparticles triggered an anti-inflammatory profile in BeWo cells, since high levels of IL-4 and IL-10 were detected. Even with regulatory profile, BeWo cells were able to control the parasites, demonstrating that the nanoparticles have other mechanisms rather than modulate the immune response.

The nanoparticles induced an anti-inflammatory profile in BeWo cells, since IL-10 is one of the most important cytokine of the maternal-fetal interface (Oliveira et al., 2006; Barbosa et al., 2008; Koga et al., 2009). Actually, there are no studies about IL-4 and *T. gondii* infection in maternal-fetal interface, the present study is to first to show it. Then, it is still unclear the role of IL-4 in trophoblast cells, regardless of the type of trophoblast involved. On the other hand, HTR8/SVneo cells produced high levels of MIF. MIF is a traditional cytokine involved during immune response against *T. gondii*, including maternal-fetal interface. Our previous studies have shown the importance of MIF in the control of parasite replication (Ferro et al., 2008; Gomes et al., 2011; Franco et al., 2014; Barbosa et al., 2015; da Silva et al., 2017). Therefore, it is not a surprise that the high levels of MIF in HTR8/SVneo cells treated with nanoparticles. It is possible to conclude that nanoparticles can act in the immune response in HTR8/SVneo cells in order to control the parasite, the opposite seen in BeWo cells. Considering all these findings, we can say that nanoparticles have different actions in dependent-manner of the trophoblast cell type.

When we investigated the AgNp-Bio action in villi, we observed that AgNp-Bio down-regulated production of IL-4, IL-6, and IL-8 in infected villi. We have to consider that villous explants are a tissue with two different populations of trophoblast, besides the mesenchyme, and then it is plausible that a different immune response can be observed. We have demonstrated in our previous studies that IL-6 is an important cytokine involved in the control of *T. gondii* (Castro et al., 2013; Barbosa et al., 2015; da Silva et al., 2017; Almeida et al., 2019), while IL-8 seems to favor the parasite replication in trophoblast cells (Milian et al., 2019). However, nanoparticles down modulated both cytokines in villous explants. Then, it is plausible to conclude that nanoparticles trigger other mechanisms to control the parasite in villi, rather than induce a protector immune response.

All these findings demonstrate that nanoparticles represent a good treatment for prevent congenital toxoplasmosis in maternal-fetal interface since they are able to reduce the parasitism without dampen the immune profile necessary to induce tolerance. In the present study, we did not verify the type of immune cells in villous explants, as other studies demonstrated in mice, for example. Chen et al. (2013) observed a dysfunction of CD4^+^CD25^+^ regulatory T cells in pregnant mice infected by *T. gondii*, inducing abortion. In our present study, we worked only with *in vitro* models. For this reason, we only observed cytokines. In a future study, it will be very interesting to investigate nanoparticles in mice models infected by *T. gondii* during pregnancy. In the next sentences, we discussed the major cytokines observed in maternal-fetal interface.

Macrophage migration inhibitory factor is a relevant cytokine in the maternal-fetal interface and is secreted by different cells types, including human trophoblast cells and human explants cells from the first and third trimester (Ferro et al., 2008; Franco et al., 2011; Gomes et al., 2011; Barbosa et al., 2014). Our group have reported the importance of MIF in congenital (Ferro et al., 2008; Gomes et al., 2011) and acquired (Flores et al., 2008; Terrazas et al., 2010) toxoplasmosis. Furthermore MIF can be associated with low recruitment of MC and decrease in indoleamine 2,3-dioxygenase (IDO) expression and consequent increase of the proinflammatory immune response (Milian et al., 2019). According to Gomes et al. (2011), the effect of MIF is dependent of age gestation. In first-trimester villous explants, MIF is increased and is able to control *T. gondii* while in third-trimester, there is a low level of MIF which can be associated to a higher vertical transmission index.

IL-6 is an important citokine in the protective immune response against infectious agents as for example, *T. gondii* (Jebbari et al., 1998; Mirpuri and Yarovinsky, 2012; Castro et al., 2013). Also, is fundamental in the process of embryo implantation and is secreted by trophoblastic cells (Goyal et al., 2013). However, IL-6 potentially can inhibit the generation of T reg cells required for pregnancy tolerance (Prins et al., 2012). Proinflammatory cytokines are necessary for implantation and vascularization, however their levels should be controlled in order to avoid gestational loss (Kalagiri et al., 2016).

In villous explants, da Silva et al. (2017) related to the reduction of proinflammatory cytokines such as IL-6 after *T. gondii* infection and treatment with enrofloxacin, toltrazuril, or combination of sulfadiazine plus pyrimethamine. According to the authors, these data may represent one strategy of the parasite to evade the immune response.

*Toxoplasma gondii* presented strategies of dissemination and survival in the host (Clough and Frickel, 2017). In the maternal-fetal interface, we verified that *T. gondii* can upregulate anti-inflammatory cytokines, such as IL-10 and TGF-β1, favoring infection of trophoblast cells (Franco et al., 2011). In general, we can infer that in normal situations of pregnancy, trophoblast cells produce anti-inflammatory cytokines such as IL-4 and IL-10 demonstrated in this study and that this may also have been enhanced by the action of AgNp-Bio in absence of *T. gondii*. Franco et al. (2011) demonstrated that treatment of human trophoblastic BeWo cells with azithromycin induces an anti-inflammatory response. Stępkowski et al. (2014) demonstrated that AgNp trigger NF-kB signaling in lung cancer cells (A549) approximately five times lower than in liver cancer cells (HepG2), which proves that cellular type influences the cellular response mediated by the NF-kB. These findings corroborate with studies by Butcher et al. (2001) demonstrating that the *T. gondii* tachyzoites avoid NF-kB activation, preventing its translocation to the core, evidencing scape mechanism of the parasite, since the induction of pro-inflammatory signaling is not activated. The same was demonstrated by Shapira et al. (2002) in fibroblasts, which proves that *T. gondii* is able to interfere in the NF-KB route escaping the immune system and fostering its survival inside the host cell. Thinking of a marked inflammatory response promoted by MIF against infection by *T. gondii*, it is important that there is a control be it immunological or by other factors that minimize this situation.

Despite all these reports, we cannot forget that there was a significant production of MIF in chorionic villi submitted to conventional treatment with the combination of sulfadiazine plus pyrimethamine. This allows us to infer that treatment with AgNp-Bio is likely to have an anti-inflammatory effect in this model. In this sense, we believe that some compounds can play this attenuating role, and specifically, in our case, it was observed that AgNp-Bio probably played such a role. The AgNp also can have an important role in the anti-inflammatory field (Zhang et al., 2016). It was also verified by Wong et al. (2009) using *in vivo* and *in vitro* models. These authors demonstrated that AgNp are able to reduce the quantity of inflammatory markers. Interestingly, David et al. (2014) detected that AgNp-Bio was also able to inhibit the production of pro-inflammatory cytokine in an immortalized lineage of keratinocytes (HaCaT). In this work, although we do not have fully elucidated data regarding the immune response to infection by *T. gondii* in these experimental models, we believe that the association of the direct and indirect action of AgNp-Bio has contributed to the inhibition of the parasite. Furthermore, according to de Araújo et al. (2019), in these experimental models, the use of AgNp allows the drug to be directed to the affected site and that the effectiveness of the transported compound is increased, in addition to suggesting a lower toxicity when compared to the conventional drug. These compounds are biocompatible, safe and tissue specific. AgNp in these experimental models can be used to determinate new therapeutic strategies against pathogens during gestation.

## Conclusion

So far, this has been a pioneering work in highlighting the anti-*T. gondii* action of AgNp synthesized in a biogenic way. The AgNp-Bio demonstrated the ability to reduce the *T. gondii* proliferation with induction of inflammatory mediators in BeWo and HTR8/SVneo cells and independent of mediators in chorionic villus. In this way, we consider the use of AgNp-Bio promising in the treatment of toxoplasmosis in BeWo and HTR8/SVneo cell models and in chorionic villi. Therefore, investigations in this sense are necessary in order to, with nanoparticles, use these experimental models be used in the evaluation of new therapeutic strategies against pathogens as *T. gondii* during pregnancy.

## Data Availability Statement

The raw data supporting the conclusions of this article will be made available by the authors, without undue reservation.

## Ethics Statement

The studies involving human participants were reviewed and approved by Ethics Committee of the Federal University of Uberlândia, MG, Brazil (Approval Number: 2.360.812). The patients/participants provided their written informed consent to participate in this study.

## Author Contributions

IC and EF designed research study. IC, MR, PS, TA, IM, LL, PG, and RS performed the experiments. IC, MR, TA, and BB analyzed the data. BB, GN, TM, JM, and EF provided the reagents. IC, PS, TA, and EF discussed the findings. IC, PS, and TA wrote the manuscript. BB and EF reviewed the manuscript. All authors contributed to the article and approved the submitted version.

### Conflict of Interest

The authors declare that the research was conducted in the absence of any commercial or financial relationships that could be construed as a potential conflict of interest.

## References

[ref1] Abou-Kheir W. Barrak J. Hadadeh O. Daoud G. (2017). HTR-8/SVneo cell line contains a mixed population of cells. Placenta 50, 1–7. 10.1016/j.placenta.2016.12.007, PMID: 28161053

[ref2] Adair B. M. (2009). Nanoparticle vaccines against respiratory viruses. Wiley Interdiscip. Rev. Nanomed. Nanobiotechnol. 1, 405–414. 10.1002/wnan.45, PMID: 20049806 PMC7169756

[ref3] Adeyemi O. S. Molefe N. I. Awakan O. J. Nwonuma C. O. Alejolowo O. O. Olaolu T.. (2018). Metal nanoparticles restrict the growth of protozoan parasites. Artif Cells Nanomed. Biotechnol. 46, S86–S94. 10.1080/21691401.2018.1489267, PMID: 30033773

[ref4] Adeyemi O. S. Murata Y. Sugi T. Kato K. (2017). Inorganic nanoparticles kill *Toxoplasma gondii* via changes in redox status and mitochondrial membrane potential. Int. J. Nanomed. 12, 1647–1661. 10.2147/IJN.S122178 PMC5339004 28280332

[ref5] Adeyemi O. S. Sulaiman F. A. (2015). Evaluation of metal nanoparticles for drug delivery systems. J. Biomed. Res. 29, 145–149. 10.7555/JBR.28.20130096 25859270 PMC4389115

[ref6] Alday P. H. Bruzual I. Nilsen A. Pou S. Winter R. Mamoun C. B. (2017). Genetic evidence for cytochrome b Qi site inhibition by 4 (1H)-quinolone-3-diarylethers and Antimycin in *Toxoplasma gondii*. Antimicrob. Agents Chemother. 61, e01866–e01816. 10.1128/AAC.01866-16 27919897 PMC5278733

[ref7] Allahverdiyev A. M. Abamor E. S. Bagirova M. Ustundag C. B. Kaya C. Kaya F., et al. (2011). Antileishmanial effect of silver nanoparticles and their enhanced antiparasitic activity under ultraviolet light. Int. J. Nanomed. 6, 2705–2714. 10.2147/IJN.S23883 PMC3218584 22114501

[ref8] Almeida M. P. O. Ferro E. A. V. Briceño M. P. P. Oliveira M. C. Barbosa B. F. Silva N. M. (2019). Susceptibility of human villous (BeWo) and extravillous (HTR-8/SVneo) trophoblast cells to *Toxoplasma gondii* infection is modulated by intracellular iron availability. Parasitol. Res. 118, 1559–1572. 10.1007/s00436-019-06257-2, PMID: 30796516

[ref9] Anbazhagan S. Azeez S. Morukattu G. Rajan R. Venkatesan K. Thangavelu K. P. (2017). Synthesis, characterization and biological applications of mycosynthesized silver nanoparticles. 3 *Biotech.* 7:333. 10.1007/s13205-017-0961-9, PMID: 28955630 PMC5603452

[ref10] Angeloni M. B. Guirelli P. M. Franco P. S. Barbosa B. F. Gomes A. O. Castro A. S. (2013). Differential apoptosis in BeWo cells after infection with highly (RH) or moderately (ME49) virulent strains of *Toxoplasma gondii* is related to the cytokine profile secreted, the death receptor Fas expression and phosphorylated ERK1/2 expression. Placenta 34, 973–982. 10.1016/j.placenta.2013.09.005 24074900

[ref11] Arokiyaraj S. Vincent S. Saravanan M. Lee Y. Oh Y. K. Kim K. H. (2017). Green synthesis of silver nanoparticles using *Rheum palmatum* root extract and their antibacterial activity against *Staphylococcus aureus* and *Pseudomonas aeruginosa*. Artif. Cells Nanomed. Biotechnol. 45, 372–379. 10.3109/21691401.2016.1160403, PMID: 27023851

[ref12] Asharani P. V. Low K. M., G., and Hande M. P. (2009). Cytotoxicity and genotoxicity of silver nanoparticles in human cells. ACS Nano. 3, 279–290. 10.1021/nn800596w, PMID: 19236062

[ref13] Assolini J. P. Concato V. M. Gonçalves M. D. Carloto A. C. M. Conchon-Costa I. Pavanelli W. R.. (2017). Nanomedicine advances in toxoplasmosis: diagnostic, treatment, and vaccine applications. Parasitol. Res. 116, 1603–1615. 10.1007/s00436-017-5458-2, PMID: 28477099

[ref14] Atilla A. Aydin S. Demirdöven A. N. Kiliç S. S. (2015). Severe toxoplasmic hepatitis in an immunocompetent patient. Jpn. J. Infect. Dis. 68, 407–409. 10.7883/yoken.JJID.2014.422, PMID: 25766609

[ref15] Balan A. Szaingurten-Solodkin I. Swissa S. S. Feinshtein V. Huleihel M. Holcberg G.. (2017). The effects of pravastatin on the normal human placenta: lessons from ex-vivo models. PLoS One 12:e0172174. 10.1371/journal.pone.0172174, PMID: 28199380 PMC5310776

[ref16] Barbosa B. F. Lopes-Maria J. B. Gomes A. O. Angeloni M. B. Castro A. S. Franco P. S., et al. (2015). IL10, TGF beta1, and IFN gamma modulate intracellular signaling pathways and cytokine production to control *Toxoplasma gondii* infection in BeWo trophoblast cells. Biol. Reprod. 92, 1–13. 10.1095/biolreprod.114.124115, PMID: 25673564

[ref17] Barbosa B. F. Paulesu L. Ietta F. Bechi N. Romagnoli R. Gomes A. O., et al. (2014). Suceptibility to *Toxoplasma gondii* proliferation in BeWo human trophoblast cells is dose-dependent of macrophage migration inhibitory factor (MIF), via ERK1/2 phosphorylation and prostaglandin E2 production. Placenta 35, 152–162. 10.1016/j.placenta.2013.1s2.013 24433846

[ref18] Barbosa B. F. Silva D. A. Costa I. N. Mineo J. R. Ferro E. A. (2008). BeWo trophoblast cell susceptibility to *Toxoplasma gondii* is increased by interferon-gamma, interleukin-10 and transforming growth factorbeta1. Clin. Exp. Immunol. 151, 536–545. 10.1111/j.1365-2249.2007.03583.x 18234060 PMC2276970

[ref19] Bernhagen J. Calandra T. Bucala R. (1998). Regulation of the immune response by macrophage migration inhibitory factor: biological and structural features. J. Mol. Med. 76, 151–161. 10.1007/s001090050204, PMID: 9535548

[ref20] Bhardwaj R. Saudagar P. Dubey V. K. (2012). Nanobiosciences: a contemporary approach in antiparasitic drugs. Mol. Cell. Pharmacol. 4, 97–103.

[ref22] Bradford M. M. (1976). A rapid and sensitive method for the quantitation of microgram quantities of protein utilizing the principle of protein-dye binding. Anal. Biochem. 72, 248–254. 10.1016/0003-2697(76)90527-3, PMID: 942051

[ref23] Butcher B. A. Kim L. Johnson P. F. Denkers E. Y. (2001). *Toxoplasma gondii* tachyzoites inhibit proinflammatory cytokine induction in infected macrophages by preventing nuclear translocation of the transcription factor NF-κB. J. Immunol. 167, 2193–2201. 10.4049/jimmunol.167.4.2193, PMID: 11490005

[ref24] Butkus M. A. Labare M. P. Starke J. A. Moon K. Talbot M. (2004). Use of aqueous silver to enhance inactivation of coliphage MS-2 by UV disinfection. Appl. Environ. Microbiol. 70, 2848–2853. 10.1128/AEM.70.5.2848-2853.2004, PMID: 15128542 PMC404450

[ref25] Carellos V. M. Andrade G. M. Q. Vasconcelos-Santos D. V. Januário J. N. Romanelli R. M. C. Abreu M. N. S.. (2014). Adverse socioeconomic conditions and oocyst-related factors are associates with congenital toxoplasmosis in a population-based study in Minas Gerais, Brazil. PLoS One 9:e88588. 10.1371/journal.pone.0088588, PMID: 24523920 PMC3921220

[ref26] Carlier Y. Truyens C. Deloron P. Peyron F. (2012). Congenital parasitic infections: a review. Acta Trop. 121, 55–70. 10.1016/j.actatropica.2011.10.018 22085916

[ref27] Castanheira L. de Souza D. L. N. Silva R. J. Barbosa B. Mineo J. R. Tudini K. A. et al. (2015). Insights into anti-parasitism induced by a C-type lectin from *Bothrops pauloensis* venom on *Toxoplasma gondii*. Int. J. Biol. Macromol. 74, 568–574. 10.1016/j.ijbiomac.2014.11.035, PMID: 25541358

[ref28] Castro A. S. Alves C. M. Angeloni M. B. Gomes A. O. Barbosa B. F. Franco P. S. (2013). Trophoblast cells are able to regulate monocyte activity to control *Toxoplasma gondii* infection. Placenta 34, 240–247. 10.1016/j.placenta.2012.12.006 23294571

[ref29] Castro-Filice L. S. Barbosa B. F. Angeloni M. B. Silva N. M. Gomes A. O. Alves C. M. O. S. (2014). Azithromycin is able to control *Toxoplasma gondii* infection in human villous explants. J. Transl. Med. 132, 1–12. 10.1186/1479-5876-12-132 PMC4039046 24885122

[ref30] Chen J. L. Ge Y. Y. Zhang J. Qiu X. Y. Qiu J. F. Wu J. P.. (2013). The dysfunction of CD4^+^CD25^+^ regulatory T cells contributes to the abortion of mice caused by *Toxoplasma gondii* excreted-secreted antigens in early pregnancy. PLoS One 8:e69012. 10.1371/journal.pone.0084522, PMID: 23874852 PMC3714236

[ref31] Ching X. T. Fong M. Y. Lau Y. L. (2016). Evaluation of Immunoprotection conferred by the subunit vaccines of GRA2 and GRA5 against acute toxoplasmosis in BALB/c mice. Front. Microbiol. 7:609. 10.3389/fmicb.2016.00609, PMID: 27199938 PMC4847622

[ref32] Clough B. Frickel E. M. (2017). The toxoplasma parasitophorous vacuole: an evolving host-parasite frontier. Trends Parasitol. 33, 473–488. 10.1016/j.pt.2017.02.007 28330745

[ref33] Costa I. N. Angeloni M. B. Santana L. A. Barbosa B. F. Silva M. C. P. Rodrigues A. A.. (2009). Azithromycin inhibits vertical transmission of *Toxoplasma gondii* in *Calomys callosus* (rodentia: cricetidae). Placenta 10, 884–890. 10.1016/j.placenta.2009.08.002, PMID: 19703714

[ref34] da Silva R. J. Gomes A. O. Franco P. S. Pereira A. S. Milian I. C. B. Ribeiro M.. (2017). Enrofloxacin and Toltrazuril are able to reduce *Toxoplasma gondii* growth in human BeWo trophoblastic cells and villous explants from human third trimester pregnancy. Front. Cell. Infect. Microbiol. 26, 7–340. 10.3389/fcimb.2017.00340, PMID: 28798905 PMC5526852

[ref35] Das V. L. Thomas R. Varghese R. T. Soniya E. V. Mathew J. Radhakrishnan E. K. (2014). Extracellular synthesis of silver nanoparticles by the Bacillus strain CS 11 isolated from industrialized area. Biotech 4, 121–126. 10.1007/s13205-013-0130-8, PMID: 28324441 PMC3964251

[ref36] David L. Moldovan B. Vulcu A. Olenic L. Perde-Schrepler M. Fischer-Fodor E.. (2014). Green synthesis, characterization and anti-inflammatory activity of silver nanoparticles using European black elderberry fruits extract. Colloids Surf. B Bioint. 122, 767–777. 10.1016/j.colsurfb.2014.08.018, PMID: 25174985

[ref37] de Araújo T. E. Milián I. C. B. de Souza G. da Silva R. J. Rosini A. M. Guirelli P. M. (2019). Experimental models of maternal–fetal interface and their potential use for nanotechnology applications. Cell Biol. Int. 44, 36–50. 10.1002/cbin.11222 31469205

[ref38] Dolati S. Sadreddini S. Rostamzadeh D. Ahmadi M. JadidiNiaragh F. Yousefi M. (2016). Utilization of nanoparticle technology in rheumatoid arthritis treatment. Biomed. Pharmacother. 80, 30–41. 10.1016/j.biopha.2016.03.004, PMID: 27133037

[ref39] Dubey J. P. Lago E. G. Gennari S. M. Su C. Jones J. L. (2012). Toxoplasmosis in humans and animals in Brazil: high prevalence, high burden of disease, and epidemiology. Parasitology 139, 1375–1424. 10.1017/S0031182012000765, PMID: 22776427

[ref40] Dunay I. R. Gajurel K. Dhakal R. Liesenfeld O. Montoya J. G. (2018). Treatment of toxoplasmosis: historical perspective, animal models, and current clinical practice. Clin. Microbiol. Rev. 31, e00057–e00017. 10.1128/CMR.00057-17 30209035 PMC6148195

[ref41] Durán N. Durán M. Jesus M. B. Seabra A. B. Fávaro W. J. Nakazato G. (2016). Silver nanoparticles: a new view on mechanistic aspects on antimicrobial activity. Nanomedicine 12, 789–799. 10.1016/j.nano.2015.11.016 26724539

[ref42] Durán N. Marcato P. D. Alves O. L. De Souza G. I. Esposito E. (2005). Mechanistic aspects of biosynthesis of silver nanoparticles by several *Fusarium oxysporum* strains. J. Nanobiotechnology 3:8. 10.1186/1477-3155-3-8 16014167 PMC1180851

[ref44] Fanti J. R. Tomiotto-Pellissier F. Miranda-Sapla M. M. Cataneo A. H. D. Andrade C. G. T. J. Panis C.. (2018). Biogenic silver nanoparticles inducing *Leishmania amazonensis* promastigote and amastigote death *in vitro*. Acta Trop. 178, 46–54. 10.1016/j.actatropica.2017.10.027, PMID: 29111137

[ref45] Ferro E. A. V. Mineo J. R. Ietta F. Bechi N. Romagnoli R. Silva D. A. O. (2008). Macrophage migration inhibitory factor is upregulated in human first-trimester placenta stimulated by soluble antigen of *Toxoplasma gondii*, resulting in increased monocyte adhesion on villous explants. Am. J. Pathol. 172, 50–58. 10.2353/ajpath.2008.070432 18165264 PMC2189627

[ref46] Flores M. Saavedra R. Bautista R. Viedma R. Tenorio E. P. Leng L.. (2008). Macrophage migration inhibitory factor (MIF) is critical for the host resistance against *Toxoplasma gondii*. FASEB J. 22, 3661–3671. 10.1096/fj.08-111666, PMID: 18606868 PMC2537436

[ref47] Foldbjerg R. Dang D. A. Autrup H. (2011). Cytotoxicity and genotoxicity of silver nanoparticles in the human lung cancer cell line, A549. Arch. Toxicol. 85, 743–750. 10.1007/s00204-010-0545-5 20428844

[ref48] Franco P. S. Gomes A. O. Barbosa B. F. Angeloni M. B. Silva N. M. Teixeira C. A. (2011). Azithromycin and spiramycin induce anti-inflammatory response in human trophoblastic (BeWo) cells infected by *Toxoplasma gondii* but are able to control infection. Placenta 32, 838–844. 10.1016/j.placenta.2011.08.012 21908042

[ref49] Franco P. S. Ribeiro M. Lopes-Maria J. B. Costa L. F. Silva D. A. Barbosa B. F., et al. (2014). Experimental infection of *Calomys callosus* with atypical strains of toxoplasma gondii shows gender differences in severity of infection. Parasitol. Res. 113, 2655–2664. 10.1007/s00436-014-3920-y, PMID: 24781027

[ref50] Gaafar M. R. Mady R. F. Diab R. G. Shalaby T. I. (2014). Chitosan and silver nanoparticles: promising anti-*Toxoplasma* agents. Exp. Parasitol. 143, 30–38. 10.1016/j.exppara.2014.05.005 24852215

[ref51] Girilal M. Fayaz A. M. Elumalai L. K. Sathiyaseelan A. Gandhiappan J. Kalaichelvan P. T. (2018). Comparative stress physiology analysis of biologically and chemically synthesized silver nanoparticles on *Solanum lycopersicum* L. Colloids Surf. B Bioint. 24, 1–6. 10.1016/j.colcom.2018.02.005

[ref52] Gomes A. O. Silva D. A. O. Silva N. M. Barbosa B. F. Franco P. S. Angeloni M. B., et al. (2011). Effect of macrophage migration inhibitory factor (MIF) in human placental explants infected with *Toxoplasma gondii* depends on gestational age. Am. J. Pathol. 178, 2792–2801. 10.1016/j.ajpath.2011.02.005, PMID: 21641401 PMC3124335

[ref53] Goyal P. Brunnert D. Ehrhardt J. Bredow M. Piccenini S. Zygmunt M. (2013). Cytokine IL-6 secretion by trophoblasts regulated via sphingosine-1-phosphate receptor 2 involving rho/rho-kinase and Rac1 signaling pathways. Mol. Hum. Reprod. 19, 528–538. 10.1093/molehr/gat023 23538947

[ref54] Gurunathan S. Han J. W. Kwon D. N. Kim J. H. (2014). Enhanced antibacterial and anti-biofilm activities of silver nanoparticles against gram-negative and gram-positive bacteria. Nanoscale Res. Lett. 9:373. 10.1186/1556-276X-9-373 25136281 PMC4127560

[ref56] Hu S. Wei W. Korner H. (2017). The role of monocytes in models of infection by protozoan parasites. Mol. Immunol. 88, 174–184. 10.1016/j.molimm.2017.06.020, PMID: 28704704

[ref57] Hussain S. M. Hess K. L. Gearhart J. M. Geiss K. T. Schlager J. J. (2005). *In vitro* toxicity of nanoparticles in BRL 3A rat liver cells. Toxicol. In Vitro 19, 975–983. 10.1016/j.tiv.2005.06.034, PMID: 16125895

[ref58] Isaac-Márquez A. P. Talamás-Rohana P. Galindo-Sevilla N. Gaitan-Puch S. E. Díaz-Díaz N. A. Hernández-Ballina G. A. (2018). Decanethiol functionalized silver nanoparticles are new powerful leishmanicidals *in vitro*. World J. Microbiol. Biotechnol. 34:38. 10.1007/s11274-018-2420-0 29460068

[ref59] Jebbari H. Roberts C. W. Ferguson D. J. Bluethmann H. Alexander J. (1998). A protective role for IL-6 during early infection with toxoplasma gondii. Parasite Immunol. 20, 231–239. 10.1046/j.1365-3024.1998.00152.x 9651924

[ref60] Jia H. Nishikawa Y. Luo Y. Yamagishi J. Sugimoto C. Xuan X. (2010). Characterization of a leucine aminopeptidase from *Toxoplasma gondii*. Mol. Biochem. Parasitol. 170, 1–6. 10.1016/j.molbiopara.2009.11.005, PMID: 19931316

[ref61] Kalagiri R. R. Carder T. Choudhury S. Vora N. Ballard A. R. Govande V. (2016). Inflammation in complicated pregnancy and its outcome. Am. J. Perinatol. 33, 1337–1356. 10.1055/s-0036-1582397 27159203

[ref62] Khalil N. M. do Nascimento T. C. F. Casa D. M. Dalmolin L. F. de Mattos A. C. Hoss I. (2013). Pharmacokinetics of curcumin-loaded PLGA and PLGA–PEG blend nanoparticles after oral administration in rats. Colloids Surf. B Bioint. 101, 353–360. 10.1016/j.colsurfb.2012.06.024 23010041

[ref63] Khanna P. Ong C. Bay B. Baeg G. (2015). Nanotoxicity: an interplay of oxidative stress, inflammation and cell death. Nano 5, 1163–1180. 10.3390/nano5031163 PMC5304638 28347058

[ref65] Kodjikian L. (2010). *Toxoplasma* and pregnancy. J. Fr. Ophtalmol. 33, 362–367. 10.1016/j.jfo.2010.03.002, PMID: 20452089

[ref66] Koga K. Aldo P. B. Mor G. (2009). Toll-like receptors and pregnancy: trophoblast as modulators of the immune response. J. Obstet. Gynaecol. 35, 191–202. 10.1111/j.1447-0756.2008.00963.x 19335792

[ref67] Larsen H. Nielsen G. L. Schønheyder H. C. Olesen C. Sørensen H. T. (2001). Birth outcome following maternal use of fluoroquinolones. Int. J. Antimicrob. Agents 18, 259–262. 10.1016/S0924-8579(01)00390-9, PMID: 11673039

[ref68] Li X. L. Wei H. X. Zhang H. Peng H. J. Lindsay D. S. (2014). A meta-analysis on risk of adverse pregnancy outcomes in *Toxoplasma gondii* infection. PLoS One 9:e97775. 10.1371/journal.pone.0097775, PMID: 24830795 PMC4022675

[ref69] Lu W. Senapati D. Wang S. Tovmachenko O. Singh A. K. Yu H.. (2010). Effect of surface coating on the toxicity of silver nanomaterials on human skin keratinocytes. Chem. Phys. Lett. 487, 92–96. 10.1016/j.cplett.2010.01.027, PMID: 24187379 PMC3812814

[ref70] Machado L. F. Sanfelicea R. A. Bosquia L. R. Assolinia J. P. Scandorieirob S. Navarro I. T.. (2020). Biogenic silver nanoparticles reduce adherence, infection, and proliferation of *Toxoplasma gondii* RH strain in HeLa cells without inflammatory mediators induction. Exp. Parasitol. 211:107853. 10.1016/j.exppara.2020.107853, PMID: 32061628

[ref71] Maenz M. Schlüter D. Liesenfeld O. Schares G. Gross U. Pleyer U. (2014). Ocular toxoplasmosis past, present and new aspects of an old disease. Prog. Retin. Eye Res. 39, 77–106. 10.1016/j.preteyeres.2013.12.005, PMID: 24412517

[ref72] Matsui M. Fowler J. H. Walling L. L. (2006). Leucine aminopeptidases: diversity in structure and function. Biol. Chem. 387, 1535–1544. 10.1515/BC.2006.191, PMID: 17132098

[ref74] Milian I. C. B. Silva R. J. Manzan-Martins C. Barbosa B. F. Guirelli P. M. Ribeiro M., et al. (2019). Increased *Toxoplasma gondii* intracellular proliferation in human Extravillous Trophoblast cells (HTR8/ SVneo line) is sequentially triggered by MIF, ERK1/2, and COX-2. Front. Microbiol. 10:852. 10.3389/fmicb.2019.00852 31068920 PMC6491458

[ref75] Miller R. K. Genbacev O. Turner M. A. Aplin J. D. Caniggia I. Huppertz B. (2005). Human placental explants in culture: approaches and assessments. Placenta 26, 439–448. 10.1016/j.placenta.2004.10.002, PMID: 15950058

[ref76] Mirpuri J. Yarovinsky F. (2012). IL-6 signaling SOCS critical for IL-12 host response to *Toxoplasma gondii*. Future Microbiol. 7, 13–16. 10.2217/fmb.11.147 22191442

[ref500] Mnkandhla D. Marwijk J. V. Hoppe H. Wilhelmi B. S. Whiteley C. G. (2018). In vivo; in vitro interaction of silver nanoparticles with leucine aminopeptidase from human and Plasmodium falciparum. J. Nanosci. Nanotechnol. 18, 865–871. 10.1166/jnn.2018.13966, PMID: 29448508

[ref77] Montazeri M. Sharif M. Sarvi S. Mehrzadi S. Ahmadpour E. Daryani A. (2017). A systematic review of *in vitro* and *in vivo* activities of anti-toxoplasma drugs na compounds. Front. Microbiol. 8:25. 10.3389/fmicb.2017.00025, PMID: 28163699 PMC5247447

[ref78] Montoya J. G. Remington J. S. (2008). Management of *Toxoplasma gondii* infection during pregnancy. Clin. Infect. Dis. 47, 554–566. 10.1086/590149 18624630

[ref80] Mosmann T. (1983). Rapid colorimetric assay for cellular growth and survival: application to proliferation and cytotoxicity assays. J. Immunol. Methods 65, 55–63. 10.1016/0022-1759(83)90303-4, PMID: 6606682

[ref81] Muoth C. Wichser A. Monopoli M. Correia M. Ehrlich N. Loeschner K. (2016). A 3D co-culture microtissue model of the human placenta for nanotoxicity assessment. Nanoscale 8, 17322–17332. 10.1039/C6NR06749B 27714104

[ref82] Oberdörster G. Oberdörster E. Oberdörster J. (2005). Nanotoxicology: an emerging discipline evolving from studies of ultrafine particles. Environ. Health Perspect. 113, 823–839. 10.3390/nano5031163 16002369 PMC1257642

[ref83] Oliveira J. G. Silva N. M. Santos A. A. Souza M. A. Ferreira G. L. Mineo J. R.. (2006). BeWo trophoblasts are unable to control replication of *Toxoplasma gondii*, even in the presence of exogenous IFN gamma. Placenta 27, 691–698. 10.1016/j.placenta.2005.06.006, PMID: 16122791

[ref84] Oz H. S. (2014). Maternal and congenital toxoplasmosis, currently available and novel therapies in horizon. Front. Microbiol. 5:385. 10.3389/fmicb.2014.00385, PMID: 25104952 PMC4109466

[ref86] Park S. Ko Y. S. Lee S. J. Lee C. Woo K. Ko G. (2018). Inactivation of influenza a virus via exposure to silver nanoparticle-decorated silica hybrid composites. Environ. Sci. Pollut. Res. Int. 25, 27021–27030. 10.1007/s11356-018-2620-z 30014367

[ref88] Petersen E. (2007). Toxoplasmosis. Semin. Fetal Neonatal Med. 12, 214–223. 10.1016/j.siny.2007.01.011 17321812

[ref89] Peyron F. McLeod R. Ajzenberg D. Contopoulos-Loannidis D. Kieffer F. Mandelbrot L.. (2017). Congenital toxoplasmosis in France and the United States: one parasite, two diverging approaches. PLoS Negl. Trop. Dis. 11:e0005222. 10.1371/journal.pntd.0005222, PMID: 28207736 PMC5312802

[ref90] Pourali P. Yahyaei B. (2016). Biological production of silver nanoparticles by soil isolated bacteria and preliminary study of their cytotoxicity and cutaneous wound healing efficiency in rat. J. Trace Elem. Med. Biol. 34, 22–31. 10.1016/j.jtemb.2015.11.004, PMID: 26854241

[ref91] Prins J. R. Gomez-Lopez N. Robertson S. A. (2012). Interleukin-6 in pregnancy and gestational disorders. J. Reprod. Immunol. 95, 1–14. 10.1016/j.jri.2012.05.004 22819759

[ref92] Rahul S. Chandrashekhar P. Hemant B. Bipinchandra S. Mouray E. Grellier P.. (2015). *In vitro* antiparasitic activity of microbial pigments and their combination with phytosynthesized metal nanoparticles. Parasitol. Int. 64, 353–356. 10.1016/j.parint.2015.05.004, PMID: 25986963

[ref93] Riehemann K. Schneider S. W. Luger T. A. Godin B. Ferrari M. Fuchs H. (2009). Nanomedicine: challenge and perspectives. Angew. Chem. Int. Ed. Eng. 48, 872–897. 10.1002/anie.200802585 PMC4175737 19142939

[ref94] Sanfelice R. A. Bosqui L. R. da Silva S. S. Miranda-Sapla M. M. Pana-Gio L. A. Navarro I. T. (2018). Proliferation of *Toxoplasma gondii* (RH strain) is inhibited by the combination of pravastatin and simvastatin with low concentrations of conventional drugs used in toxoplasmosis. J. Appl. Biomed. 16, 29–33. 10.1016/J.jab.2017.10.009

[ref95] Sanfelice R. A. Machado L. F. Bosqui L. R. Miranda-Sapla M. M. Tomiotto-Pellissier F. De Alcântara D. G.. (2017). Activity of rosuvastatin in tachyzoites of *Toxoplasma gondii* (RH strain) in HeLa cells. Exp. Parasitol. 181, 75–81. 10.1016/j.exppara.2017.07.009, PMID: 28774497

[ref96] Sanguiñedo P. Fratila R. M. Estevez M. B. De La Fuente J. M. Grazú V. Alborés S. (2018). Extracellular biosynthesis of silver nanoparticles using fungi and their antibacterial activity. Nano Biomed. Eng. 10, 156–164. 10.5101/nbe.v10i2.p165-173

[ref97] Scandorieiro S. De Camargo L. C. Lancheros C. A. Yamada-Ogatta S. F. Nakamura C. V. De Oliveira A. G. (2016). Synergistic and additive effect of oregano essential oil and biological silver nanoparticles against multidrug-resistant bacterial strains. Front. Microbiol. 7:760. 10.3389/fmicb.2016.00760 27242772 PMC4876125

[ref98] Sepúlveda-Arias J. C. Veloza L. A. Mantilla-Muriel L. E. (2014). Anti-*Toxoplasma* activity of natural products: a review. Recent Pat. Antiinfect. Drug Discov. 9, 186–194. 10.2174/1574891x10666150410120321, PMID: 25858302

[ref99] Shapira S. Speirs K. Gerstein A. Caamano J. Hunter C. A. (2002). Suppression of NF-κB activation by infection with *Toxoplasma gondii*. sis. 185, S66–S72. 10.1086/338000 11865442

[ref100] Sharma V. Kaushik S. Pandit P. Dhull D. Yadav J. P. Kaushik S. (2019). Green synthesis of silver nanoparticles from medicinal plants and evaluation of their antiviral potential against chikungunya virus. Appl. Microbiol. Biotechnol. 103, 881–891. 10.1007/s00253-018-9488-1 30413849

[ref101] Shiono Y. Mun H. S. He N. Nakazaki Y. Fang H. Furuya M. (2007). Maternal-fetal transmission of *Toxoplasma gondii* in interferon-gamma deficient pregnant mice. Parasitol. Int. 56, 141–148. 10.1016/j.parint.2007.01.008 17307382

[ref102] Shrivastava S. Bera T. Roy A. Singh G. Ramachandrarao P. Dash D. (2007). Characterization of enhanced antibacterial effects of novel silver nanoparticles. Nanotechnology 18:225103. 10.1088/0957-4484/18/22/225103 37016550

[ref103] Stępkowski T. M. Brzóska K. Kruszewski M. (2014). Silver nanoparticles induced changes in the expression of NF-κB related genes are cell type specific and related to the basal activity of NF-κB. Toxicol. In Vitro 28, 473–478. 10.1016/j.tiv.2014.01.008 24462830

[ref104] Sutterland A. L. Fond G. Kuin A. Koeter M. W. J. Lutter R. Van Gool T. (2015). Beyond the association *Toxoplasma gondii* in schizophrenia, bipolar disorder, and addiction: systematic review and meta-analysis. Acta Psychiatr. Scand. 132, 161–179. 10.1111/acps.12423 25877655

[ref105] Terrazas C. A. Juarez I. Tettazas L. I. Saavedra R. Calleja E. A. Rodriguez-Sosa M. (2010). *Toxoplasma gondii*: impaired maturation and pro-inflammatory response of dendritic cells in MIF-deficient mice favors susceptibility to infection. Exp. Parasitol. 126, 348–358. 10.1016/j.exppara.2010.03.009 20331989

[ref106] Torres-Santiago E. Holban A. Gestal M. (2016). Advanced nanobiomaterials: vaccines, diagnosis and treatment of infectious diseases. Molecules 21:867. 10.3390/molecules21070867 PMC6273484 27376260

[ref107] Valero L. Alhareth K. Gil S. Lecarpentier E. Tsatsaris V. Mignet N.. (2018). Nanomedicine as a potential approach to empower the new strategies for the treatment of preeclampsia. Drug Discov. Today 23, 1099–1107. 10.1016/j.drudis.2018.01.048, PMID: 29391261

[ref108] Velhal S. G. Kulkarni S. D. Latpate R. V. (2016). Fungal mediated silver nanoparticle synthesis using robust experimental design and its application in cotton fabric. Int Nano Lett. 6, 257–264. 10.1007/s40089-016-0192-9

[ref109] Wong K. K. Y. Cheung S. O. F. Huang L. Niu J. Tao C. Ho C. M.. (2009). Further evidence of the anti-inflammatory effects of silver nanoparticles. ChemMedChem 4, 1129–1135. 10.1002/cmdc.200900049, PMID: 19405063

[ref110] Yarovinsky F. (2014). Innate immunity to *Toxoplasma gondii* infection. Nat. Rev. Immunol. 14, 109–121. 10.1038/nri3598 24457485

[ref111] Zhang X. F. Liu Z. G. Shen W. Gurunathan S. (2016). Silver nanoparticles: synthesis, characterization, properties, applications, and therapeutic approaches. Int. J. Mol. Sci. 17, 1534. 10.3390/ijms17091534 PMC5037809 27649147

[ref112] Zheng J. Wu X. Wang M. Ran D. Xu W. Yang J. (2008). Study on the interaction between silver nanoparticles and nucleic acids in the presence of cetyltrimethylammonium bromide and its analytical application. Talanta 74, 526–532. 10.1016/j.talanta.2007.06.014, PMID: 18371671

